# Integrative “omic” analysis reveals distinctive cold responses in leaves and roots of strawberry, *Fragaria* × *ananassa* ‘Korona’

**DOI:** 10.3389/fpls.2015.00826

**Published:** 2015-10-15

**Authors:** Gage Koehler, Jens Rohloff, Robert C. Wilson, Joachim Kopka, Alexander Erban, Per Winge, Atle M. Bones, Jahn Davik, Muath K. Alsheikh, Stephen K. Randall

**Affiliations:** ^1^Department of Biology, Indiana University–Purdue University Indianapolis, IndianapolisIN, USA; ^2^Department of Biology, Norwegian University of Science and TechnologyTrondheim, Norway; ^3^Department of Natural Sciences and Technology, Hedmark University CollegeHamar, Norway; ^4^Max Planck Institute of Molecular Plant PhysiologyPotsdam, Germany; ^5^Bioforsk, Norwegian Institute for Agricultural and Environmental Research – Grassland and Landscape DivisionKvithamar, Norway; ^6^Graminor Breeding Ltd.Ridabu, Norway; ^7^Department of Plant Sciences, Norwegian University of Life SciencesÅs, Norway

**Keywords:** *Fragaria*, Korona, cold, acclimation, proteome, transcriptome, metabolome, tolerance

## Abstract

To assess underlying metabolic processes and regulatory mechanisms during cold exposure of strawberry, integrative “omic” approaches were applied to *Fragaria* × *ananassa* Duch. ‘Korona.’ Both root and leaf tissues were examined for responses to the cold acclimation processes. Levels of metabolites, proteins, and transcripts in tissues from plants grown at 18°C were compared to those following 1–10 days of cold (2°C) exposure. When leaves and roots were subjected to GC/TOF-MS-based metabolite profiling, about 160 compounds comprising mostly structurally annotated primary and secondary metabolites, were found. Overall, ‘Korona’ showed a modest increase of protective metabolites such as amino acids (aspartic acid, leucine, isoleucine, and valine), pentoses, phosphorylated and non-phosphorylated hexoses, and distinct compounds of the raffinose pathway (galactinol and raffinose). Distinctive responses were observed in roots and leaves. By 2DE proteomics a total of 845 spots were observed in leaves; 4.6% changed significantly in response to cold. Twenty-one proteins were identified, many of which were associated with general metabolism or photosynthesis. Transcript levels in leaves were determined by microarray, where dozens of cold associated transcripts were quantitatively characterized, and levels of several potential key contributors (e.g., the dehydrin COR47 and GADb) to cold tolerance were confirmed by qRT-PCR. Cold responses are placed within the existing knowledge base of low temperature-induced changes in plants, allowing an evaluation of the uniqueness or generality of *Fragaria* responses in photosynthetic tissues. Overall, the cold response characteristics of ‘Korona’ are consistent with a moderately cold tolerant plant.

## Introduction

Plants are constantly exposed to challenging abiotic stresses, such as temperature and water availability. Cold temperatures are a challenge plants commonly encounter in the temperate and sub-polar regions of the world. While we are still at the first steps in understanding the interactions between a suite of interacting low-temperature regulatory transcription factors (Park et al., 2015) a major contributor to cold hardening and adaptation to low temperatures is the activation of the CBF regulon (Stockinger et al., 1997; Vogel et al., 2006). The CBF response pathway is highly characterized in *Arabidopsis* and has been reported to occur in many crop plants (Yang et al., 2005) including strawberry (Owens et al., 2002). In *Arabidopsis thaliana*, following a partially characterized cold-induced signal cascade, the immediate activation of the ICE1 transcription factor (Zarka et al., 2003) and CAMTA (Doherty et al., 2009; Kim et al., 2013) is followed by induction and accumulation of the CBF transcription factors and the subsequent activation of the CBF regulon (Lee et al., 2005). Following activation of the CBF regulon (comprised of 100s of genes), the plant undergoes many physiological and molecular changes that affect both primary and secondary metabolism. These mechanisms include the functional expression of hydrophilic and cryoprotective proteins (Alsheikh et al., 2003, 2005), and the metabolic regulation of low-molecular weight compounds which act as osmolytes and osmoprotectants (Vogel et al., 2006; Kaplan et al., 2007; Guy et al., 2008). In cold tolerant plants these processes lead to enhanced stress-tolerance.

The cold response and freezing tolerance of perennial crops is of major interest for breeders and farmers in temperate and sub-polar climate zones due to short vegetation periods and harsh climatic conditions. An important horticultural crop for the consumer market is the cultivated strawberry (*Fragaria* × *ananassa* Duch.). Successful production and berry yield relies mainly on the plants acclimation winter survival, and rapid re-growth in spring time (Rohloff et al., 2009). Although responses to environmental stresses are known to be highly evolutionary, conserved throughout the plant kingdom (Ruelland et al., 2009), one might expect different cold acclimation and freezing tolerance strategies utilized in annual (e.g., *Arabidopsis*) and perennial species (e.g., strawberry).

Cold-regulated transcripts have been identified in *F. ananassa* leaves (Ndong et al., 1997; Yubero-Serrano et al., 2003; Koehler et al., 2012a). Further, levels of cold-regulated proteins in crowns of several cultivars of *F. ananassa* varying in cold tolerance have been evaluated (Koehler et al., 2012b). Several varieties of *F. vesca* were analyzed for metabolite changes in response to cold acclimation (Rohloff et al., 2012). However, a coordinate multi-omic analysis had not been conducted. The value of such a meta-analysis is not only to identify functional responses that might contribute to cold tolerance and hence be utilized as important molecular markers for breeding purposes; but also to provide a background for understanding the depth of regulatory mechanisms involved in cold acclimation. In addition, a comparison of metabolic changes that occur following cold acclimation in leaves and in roots of the octaploid *F. ananassa*, ‘Korona’ was obtained.

The purpose of this study was to (1) compare the cold-induced changes in metabolites in root and leaves, (2) to analyze the leaf metabolite changes with respect to those changes occurring at the protein and transcriptional levels, and (3) to evaluate whether marker analysis of above ground tissues (leaves) could substitute for subterranean tissues (roots or crowns) in marker analysis. The strawberry cultivar ‘Korona,’ used in this study, was originally developed in the Netherlands and is often cultivated in challenging climates of both Central Europe and Scandinavia where, by anecdotal data (from farmers) and unpublished controlled freezing damage estimates (Muath K. Alsheikh, personal communication); it shows cold tolerance intermediate between that of Elsanta (weak cold tolerance) and Jonsok (strongest cold tolerance) cultivars (Koehler et al., 2012b).

## Materials and Methods

### Plant Growth and Cold Treatment

Eight weeks old runner-propagated *Fragaria* × *ananassa* Duch. ‘Korona,’ were grown on fertilized soil (P-Jord; Emmaljunga Torvmull AB) in plug trays (3 × 6 cells) in a greenhouse at 18°C under natural light and long-day (16 h) conditions. To slowly adapt plants to above-zero cold temperatures, plants were short-day (12 h) adapted for 1 week at 10°C under artificial light (fluorescent tubes, ∼90 μmol m^-2^ sec^-1^) in a conditioning room prior to transfer to a cold storage room at 2°C under artificial light (short days, fluorescent tubes, ∼90 μmol m^-2^ sec^-1^) and relative humidity at average of 80%. Plants were watered before placing at 2°C and then were watered as needed to keep the soil moist over the 10 days. Plant sampling was carried out at times after initiation of the cold treatment. Control samples (0 h) were harvested prior to the transfer to the cold room. Harvested plant material of leaves or roots from three plants per time point was pooled, flash-frozen in liquid N_2_, and stored at –80°C before sample processing. Samples were taken at 0, 3, 24, 72, and 240 h after acclimation at 2°C. All samples were taken at ZT4 (4 h after lights came on) except for the 3-h point (at ZT7). For metabolite analysis all time points were analyzed; for protein 0, 24, and 240 h were analyzed; and for microarray 0, 24, and 72 h were examined. Both leaf and root were analyzed for metabolite profiles, while only leaves were utilized for protein and transcript analysis.

### Metabolite Analysis

#### Sample Extraction and Metabolite Profiling

Metabolite profiling was essentially as described previously (Rohloff et al., 2012). In detail, samples of 120 mg frozen fresh weight were transferred from pooled material into round-bottomed 1.5 ml microfuge tubes and homogenized using an oscillating ball mill while kept frozen. Frozen powder was mixed with 360 μl pre-cooled methanol containing ribitol as internal standard for the correction of volume errors. Samples were extracted at 70°C for 15 min. After cooling to room temperature 200 μl CHCl_3_ were added to the tubes which were then agitated at 37°C for 5 min. Finally, 400 μl H_2_O were added in order to induce liquid phase separation. Samples were vortexed prior to centrifugation at 14,000 rpm for 5 min. Eighty microliter of the upper polar phase containing fraction enriched for primary metabolites was transferred into a 1.5 ml tapered microfuge tube, dried in a SpeedVac vacuum concentrator overnight without heating, and stored dry at –80°C. Chemical derivatization, i.e., methoxyamination and trimethylsilylation, and subsequent GC/TOF-MS based metabolite profiling was as described by Sanchez et al. (2008).

#### Metabolite Data Processing and Analysis

Chromatographic data sets were aligned and baseline corrected using the MetAlign software (Lommen, 2009). TagFinder software v.4.0 (Luedemann et al., 2008) was used for subsequent non-targeted, multi-parallel chromatography data processing, data matrix generation, and metabolite identification using authenticated reference spectra from the Golm Metabolome Database (Kopka et al., 2005; Hummel et al., 2007). Numerical analysis was based on peak height values (response) which were corrected for fresh weight variation and using the internal standard ribitol.

Prior to statistical assessment, log-transformed response ratios [log_2_(N)] were calculated for each of the 160 identified metabolites and non-identified mass spectral tags of leaf and root metabolite profiles. Ratios were based on the median of the initial time point (t0, *n* = 5) of all genotypes, and used for Principal Component Analysis (PCA). Graphical processing and statistical analyses, such as hierarchical clustering using the distance measure, Pearson’s correlation, and complete linkage was performed with the MultiExperiment Viewer software v.4.4 (Saeed et al., 2003). Venn diagrams were drawn with Microsoft^®^ Word, and are based on 160 identified metabolites and non-identified mass spectral tags from all time points. Furthermore, metabolite data were averaged into the early (means of 3 and 24 h time points) and late (means of 72 and 240 h time points) metabolic phenotype. The threshold for considering differentially regulated metabolites was [log_2_(N)] (response ratio) ≤ 0.25 or ≥ –0.25. Functional, unweighted network diagrams of early (means of 3 and 24 h time points) and late (means of 72 and 240 h time points) metabolic response to cold treatment in leaf and root samples of *Fragaria* genotypes were drawn using the Pajek software v. 1.24 (Batagelj and Mrvar, 1998). The metabolites of the network study (**Figure 4**) were chosen from the list of 61 compounds that include pathway-associated compounds. Non-identified metabolites were excluded from this analysis.

### Proteomic Analysis

#### Protein Extraction, 2DE (Two-Dimensional Gel Electrophoresis), and Analysis

Tissues (from 0, 24, and 240 h cold acclimation) were ground to a fine powder in liquid nitrogen in the presence of polyvinylpolypyrrolidone (PVPP) at 10% of tissue weight. The powder was further processed for 2DE exactly as previously described (Koehler et al., 2012b). First dimension (isoelectric focusing, pH 3–10 non-linear) and second dimension (12% SDS-PAGE) were run and processed as previously described (Koehler et al., 2012b). Colloidal coomassie stained gels (Candiano et al., 2004) were imaged with a Molecular Imager FX Laser-Based Scanner and PDQuest version 7.1 (Bio-Rad Laboratories, Hercules, CA, USA) was used to evaluate nine (three conditions, each in triplicate) 2DE gels. A total of 845 total protein spots were matched and inspected visually to validate all automated matching. The protein spot quantities were normalized based on the total valid spots for each gel and expressed as parts per million (ppm). Average intensities, standard deviations and coefficient of variations were obtained. Significant protein spot differences were determined between time points using Student’s *t*-test (unpaired, two tailed) *p* < 0.05.

#### 2DE Protein Identification by LC–MS/MS

The gel spots were manually cut from the wet gels. The gel plugs were destained with 50% acetonitrile (ACN) in 50 mM ammonium bicarbonate (NH_4_HCO_3_) twice, reduced with 10 mM DTT in 100 mM NH_4_HCO_3_, alkylated with 55 mM iodoacetamide in 100 mM NH_4_HCO_3_, and digested by trypsin for 3 h at 37°C. The tryptic peptides were extracted with 30, 50, and 100% ACN sequentially. The extracted peptides combined were dried by SpeedVac and reconstituted with 5% ACN in 0.1% FA (formic acid). The peptide samples were analyzed using a Thermo–Finnigan linear ion-trap (LTQ) mass spectrometer coupled with a Surveyor autosampler and MS HPLC system (Thermo–Finnigan). Tryptic peptides were injected onto the C18 microbore RP column (Zorbax SB-C18, 1.0 mm × 50 mm) at a flow rate of 50 μL/min. The mobile phases A, B, and C were 0.1% FA in water, 50% ACN with 0.1% FA in water, and 80% ACN with 0.1% FA in water, respectively. The gradient elution profile was as follows: 10% B (90% A) for 10 min, 10–20% B (90–80% A) for 5 min, 20–70% B (80–30% A) for 35 min, and 100% C for 10 min. The data were collected in the “Data dependent MS/MS” mode with the ESI interface using the normalized collision energy of 35%. Dynamic exclusion settings were set to repeat count 2, repeat duration 30 s, exclusion duration 120 s, and exclusion mass width 1.50 m/z (low) and 1.50 m/z (high). The acquired tandem mass spectrometry data were processed using Trans-Proteomic Pipeline (TPP) software v. 4.8.0 [developed by the Institute for Systems Biology (ISB) in the Seattle Proteome Center], http://tools.proteomecenter.org/software.php. The workflow consisted of converting raw data to mzML file and then using the default search parameters using the accurate mass binning setting. The X!Tandem search was done against the Rosacea reference proteome set (NCBI 2014_09) and the *F. vesca* reference set (NCBI 2014_09). The peptides and proteins were validated by PeptideProphet and ProteinProphet and filtered using the following cut-offs: (1) the confidence of protein was ≥99.00% (0.9000); (2) at least two peptides were identified for a protein; and (3) the confidence of peptides was ≥80.00% (0.8000) with at least one peptide’s confidence ≥90.00% (0.9000). Only the peptides and proteins meeting the above criteria are shown.

#### Western Blotting

Equal amounts of protein from total extracts (from cold acclimations of 0, 24, and 240 h) were separated by 10% one-dimensional PAGE, transferred to nitrocellulose and probed with antibody raised against *Arabidopsis* COR47 as previously described (Alsheikh et al., 2005).

### Transcriptional Analysis

#### Transcriptional Profiling based on *Arabidopsis* Oligonucleotide Microarrays

Fifty milligram of leaf tissue from time points 0 (t0), 24, and 72 h after cold treatment was kept in 2 ml tubes in a 24-well adapter at –80°C before disrupting in a TissueLyser (Qiagen) using 5 mm stainless steel beads (2 min at 25 Hz). RNA was subsequently extracted using Spectrum^TM^ Plant Total RNA kit (Sigma–Aldrich). After adding 500 μL lysis buffer to samples, tubes were transferred to a TissueLyser adapter at ambient temperature and homogenized for 2 min at 25 Hz. Samples were kept at room temperature for 4 min before cell debris was spun down at 13,000 rpm, and supernatant transferred to a new tube. This step was repeated twice before sample was loaded onto filtration columns. To improve RNA purity, a subsequent RNA clean up procedure was performed. Four RNA isolates were pooled and concentrated on Microcon^®^ YM30 columns (Millipore) to a final volume of 50 μL. Then 500 μL lysis buffer was added and samples incubated for 4 min at 56°C, before adding 500 μL binding buffer and loading onto the binding column. Eluted RNA was concentrated on Microcon^®^ YM30 columns (Millipore) to a final volume of 20 μL. RNasin^®^ (Promega) was added to a final concentration of 1 u/μl to protect RNA from degradation. RNA quantity and purity was analyzed on a Nanodrop spectrophotometer (Nanodrop^®^ Technologies), and RNA integrity was analyzed by formaldehyde agarose gel electrophoresis.

Total RNA samples from each time point, all consisting of four independent biological replicates, were labeled using a 3DNA Array 350 kit with Cy3- and Cy5-labeled dendrimers (Genisphere Inc., Hatfield, PA, USA). Super-Script III reverse transcriptase (Invitrogen, Carlsbad, CA, USA) were used for reverse transcription. To avoid dye-bias artifacts, Cy3- and Cy5-labeled samples were swapped in individual hybridizations. The *A. thaliana* 34K NARC series 8 (GPL11051) microarray chip was used in all hybridizations. Hybridizations were performed using an Advalytix ArrayBooster^TM^ hybridization station. Hybridization and washing temperatures were reduced to 55°C which allows for mismatches between oligo nucleotide probes (designed for *A. thaliana*) and *F. vesca* transcripts. The slides were scanned at 10 mm resolution on a G2505B Agilent DNA microarray scanner (Agilent Technologies, Palo Alto, CA, USA) and images were processed using GenePix 5.1 software (Axon Instruments, Union City, CA, USA).

Statistical analyses were performed using the Limma package (Smyth, 2004), signals were log-transformed and normalized using the print-tip loess normalization method. Control spikes and landmarks (Cy3 and Cy5 oligos) were excluded from normalization, no background subtraction were performed. These data were further assessed by comparison of *Arabidopsis* oligo nucleotide probes with published *F. vesca* genome sequences using tBLASTx. More detailed method information is described in Kusnierczyk et al. (2008). The raw data can be accessed at NCBI/GEO accession GSE73488; http://www.ncbi.nlm.nih.gov/geo/query/acc.cgi?acc=GSE73488.

#### Quantitative Real-time RT-PCR

Total RNA from cold treated samples (time points 0, 24, and 72 h) employed in the microarray analyses was used for qRT-PCR. Approximately 2 μg total RNA of each sample was treated with Amplification Grade DNase I (Sigma–Aldrich, St. Louis, MO, USA) according to manufacturer’s instructions, then divided evenly between two PCR plates that served as technical replicates. Reverse transcription (RT) of the samples in each replicate plate was performed independently using oligo-dT12-18 primers and the SuperScript III First-Strand Synthesis System (Invitrogen, Carlsbad, CA, USA) according to the manufacturer’s instructions. To control for any non-degraded (Smyth, 2004), contaminating genomic DNA, selected replicate samples were included on each plate in which H_2_O replaced Superscript III in the RT reactions. Each replicate set of cDNAs were independently used as templates for qPCR with gene-specific primers designed to produce amplicons between 150 to 450 bp in size. Targeted transcripts included COR47 (positive control gene for cold acclimation), F3H (flavonone-3-hydroxylase), GADb (glutamate decarboxylase, ortholog b), and MIPS (myo-inositol-1-phosphate synthase 3). Transcript levels were normalized to PP2A (protein phosphatase 2A), a superior reference gene for transcript normalization in studies of abiotic stress in *Arabidopsis* [96]. SYBR Green Power Master Mix and the 7500 Real-Time PCR System (Applied Biosystems, Carlsbad, CA, USA) was used for the qPCR. Each gene-specific primer pair (**Supplementary File S4**) generated a single, expected PCR product based on post-PCR dissociation analyses, agarose gel electrophoresis and direct sequencing of the PCR products using BigDye terminator chemistry (v3.1, Applied Biosystems). Fold expression in cold treated samples were calculated relative to the 0 h time point for the corresponding genotype (expression levels set to 1) according to method of Pfaﬄ (2001).

## Results

### Metabolic Responses during Cold Acclimation

**FIGURE 1:**
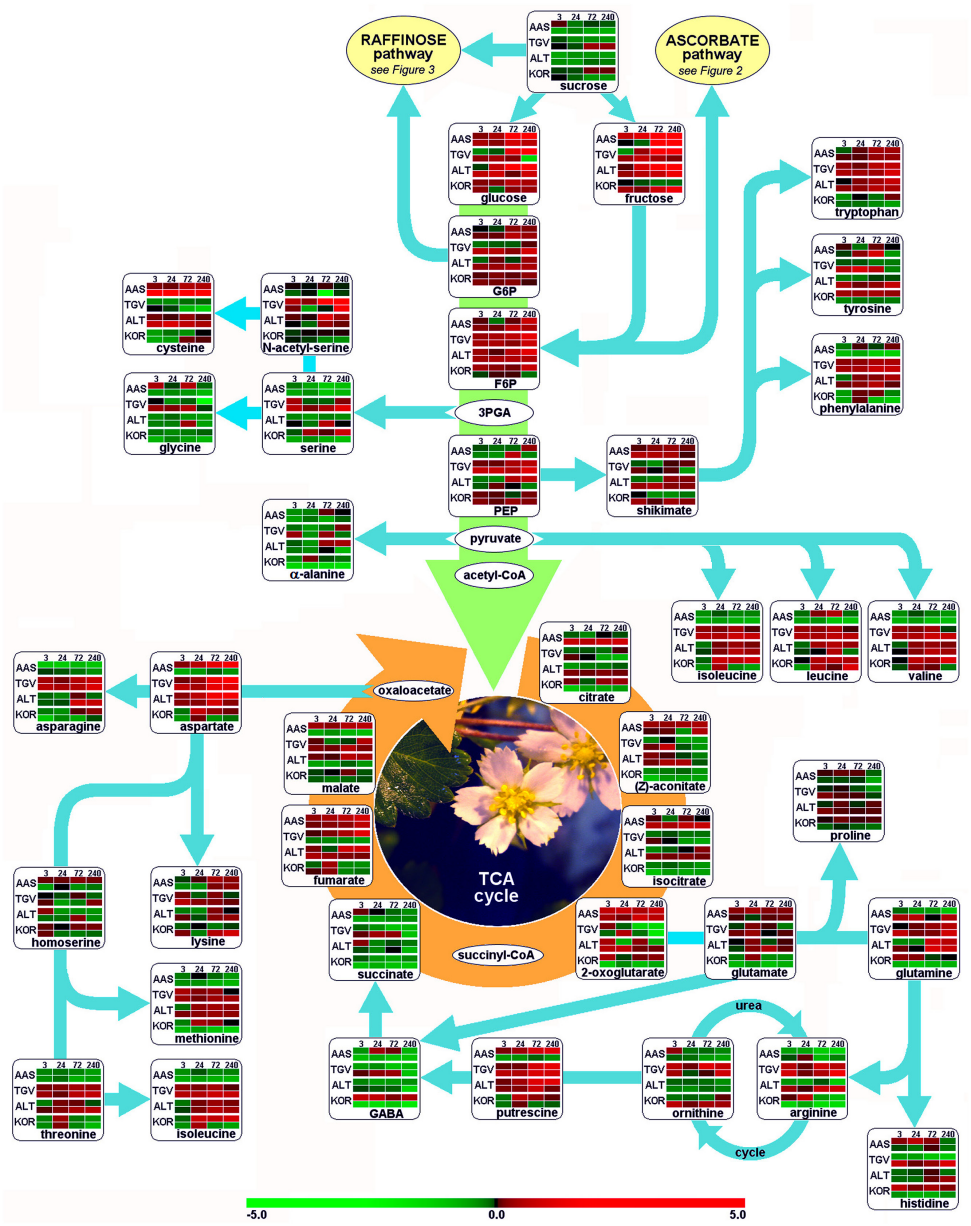
**Functional regulation of primary metabolism.** Glycolysis, citric acid cycle (TCA), and amino acid biosynthesis are depicted as pathway map. Leaf and root samples of *Fragaria x ananassa* (KOR: cv. ‘Korona’) harvested at different time points after cold treatment (0, 3, 24, 72, and 240 h), are compared to those of the diploid *F. vesca* cultivars; AAS (cv. Ås, Southern, continental Norway), TGV (cv. Tingvoll, coastal Mid-Norway) and ALT (cv. Alta, Northern Norway). Colors in metabolite arrays represent ratio values based on the median of the initial time point (t0, *n* = 5) of each genotype × plant organ combination. The upper row from paired rows of each genotype represents leaf samples, the lower row the roots. Green colors indicate down-regulation of metabolites, red colors up-regulation (see color scale). Metabolite colors (intensities) were generated using the MultiExperiment Viewer software v.4.4 (Saeed et al., 2003). 3PGA, 3-phosphoglyceraldehyde; F6P, fructose-6-phosphate; G6P, glucose-6-phosphate; GABA, 4-aminobutyric acid; PEP, phosphoenolpyruvate.

**FIGURE 2:**
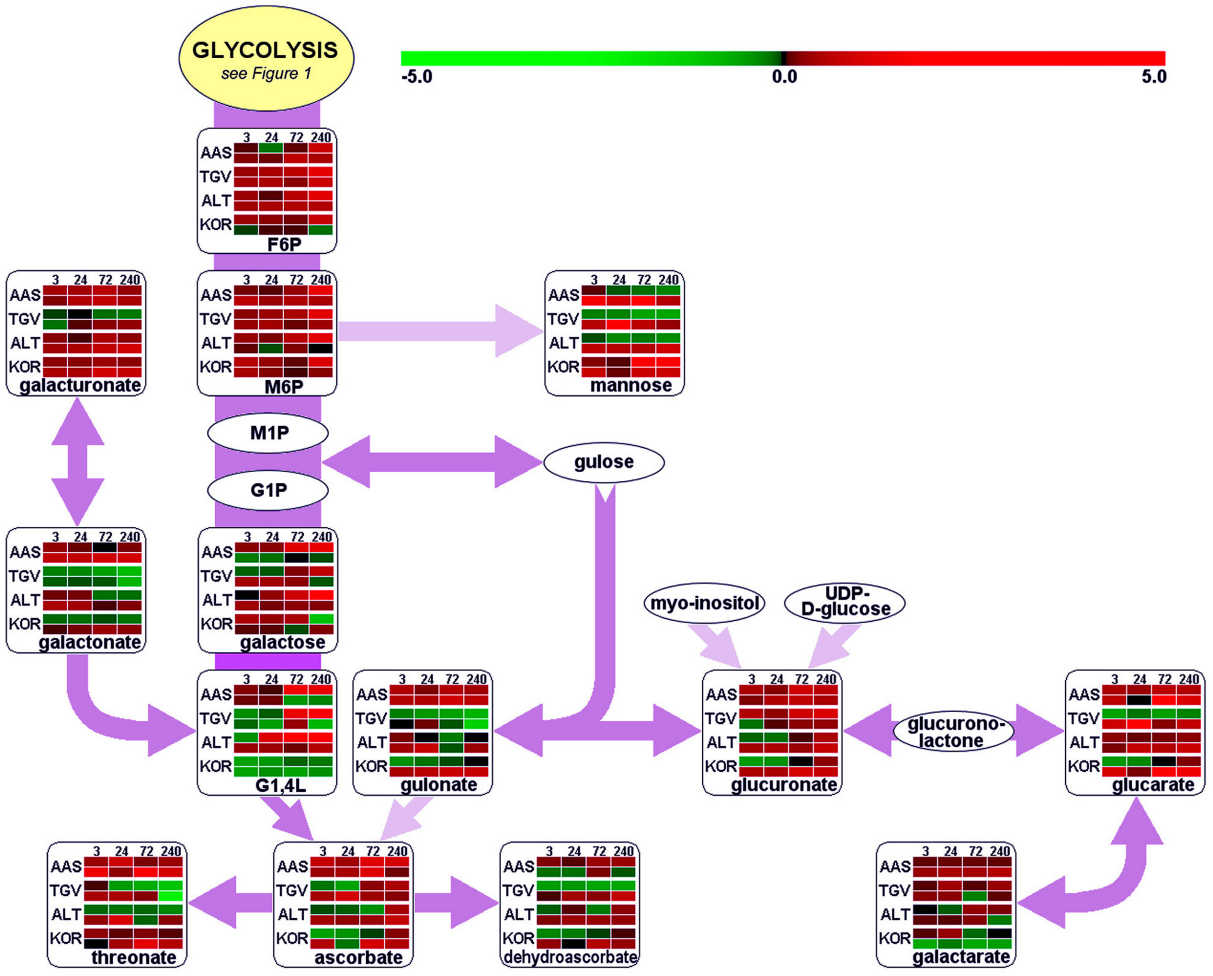
**Functional regulation of ascorbate metabolism.** For further details about pathway map, experiments and color settings, see **Figure 1**. F6P, fructose-6-phosphate; G1,4L, galactonic acid-1,4-lactone; G1P, galactose-1-phosphate; M1P, mannose-1-phosphate; M6P, mannose-6-phosphate; UDP, uridine-diphosphate.

**FIGURE 3:**
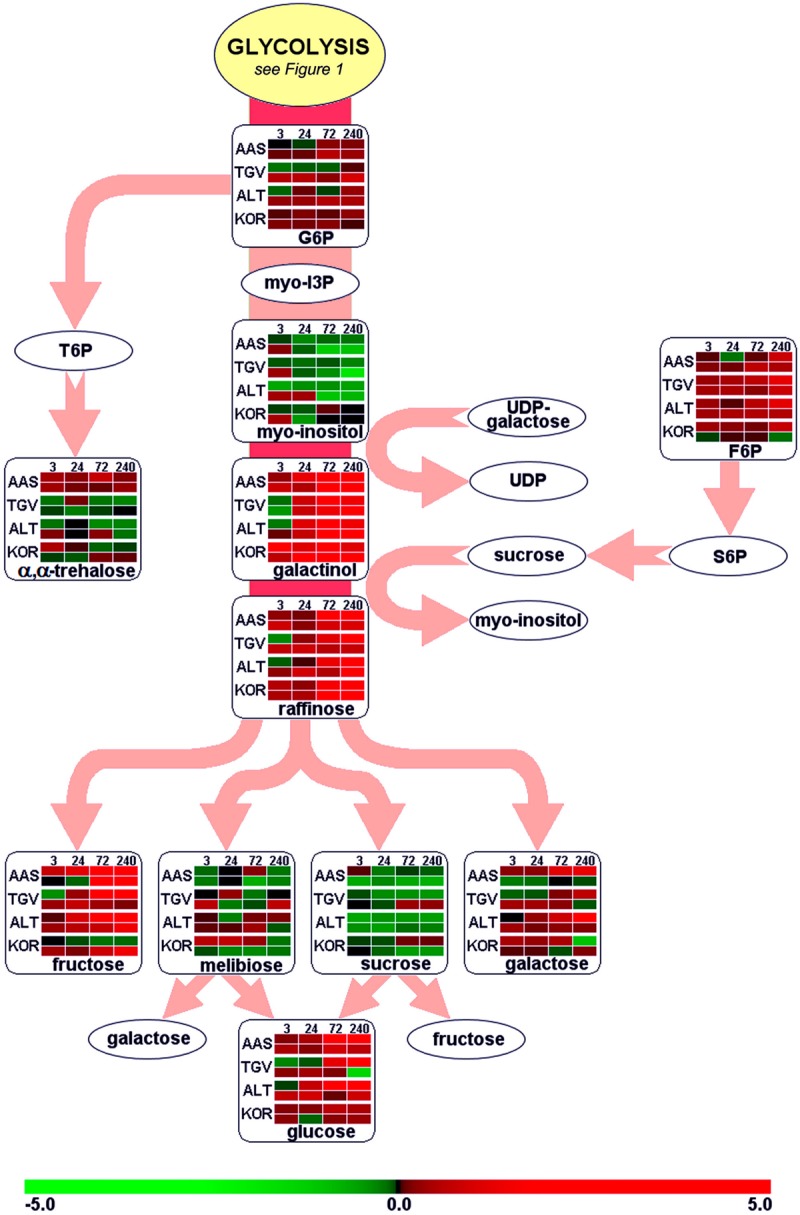
**Functional regulation of raffinose biosynthesis.** For further details about pathway map, experiments and color settings, see **Figure 1**. F6P, fructose-6-phosphate; G6P, glucose-6-phosphate; myo-I3P, myo-inositol-3-phosphate; S6P, sucrose-6-phosphate; T6P, trehalose-6-phosphate; UDP, uridine-diphosphate.

**FIGURE 4:**
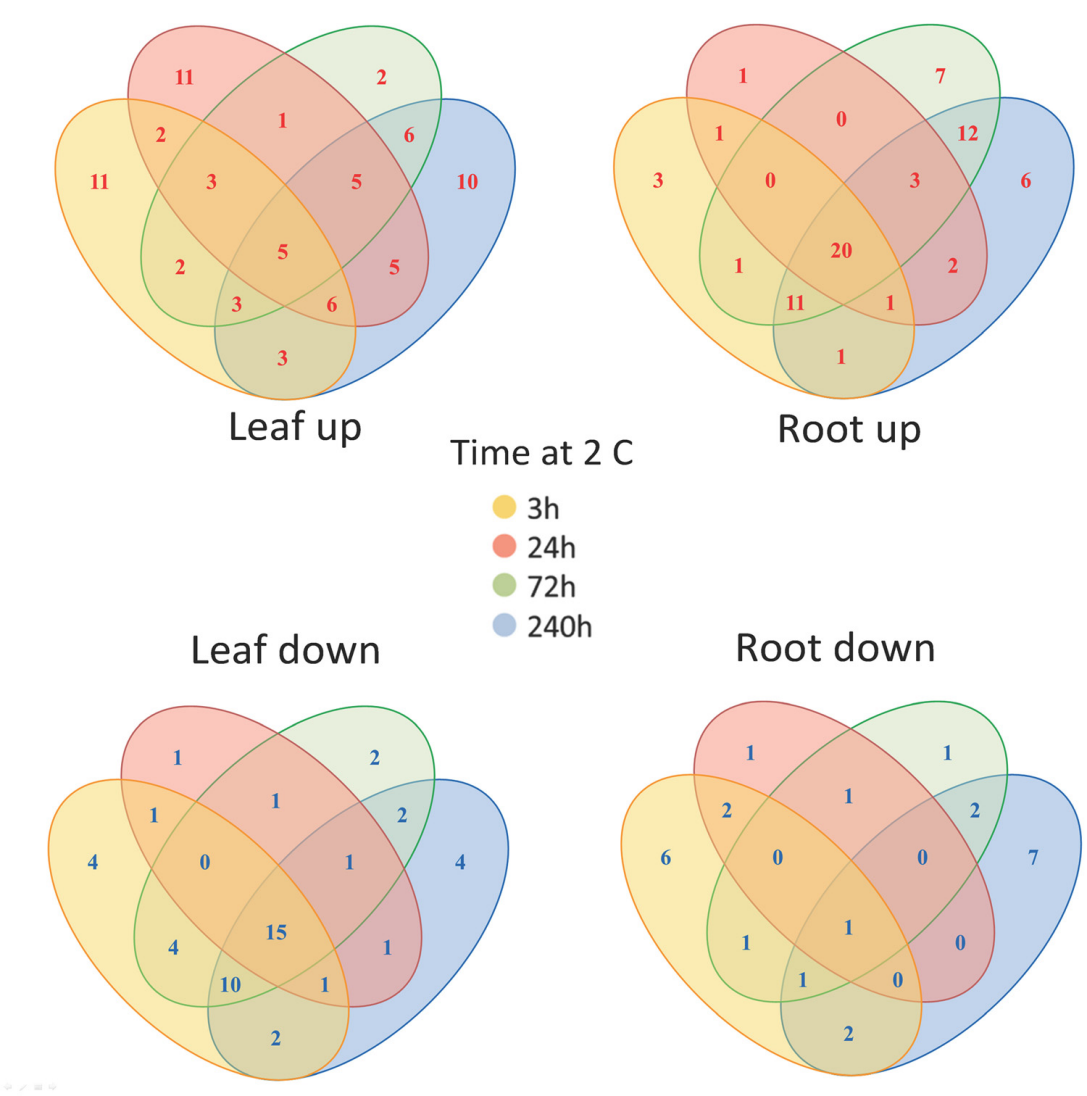
**Changes in metabolite levels in leaves and roots following cold treatment.** The Venn diagrams show the co-ordinate UP or DOWN regulation of metabolism based on averaged data (160 identified metabolites and non-identified mass spectral tags) at the various time points.

Leaves and roots from *F. ananassa* ‘Korona’ plants, following 0, 3, 24, 72, or 240 h of cold-exposure, were subjected to GC/TOF-MS-based metabolic profiling. A total of 160 compounds comprising both structurally annotated primary metabolites (129 compounds) and as yet non-identified mass spectral tags, i.e., metabolic components recognized by mass spectrum and retention index, were detected (**Supplementary File S1**). The results showed dynamic changes occurring in both leaves and roots as a function of time with relatively few metabolites showing sustained changes throughout the time course. As a summary, distinct identified compounds regulated upon cold acclimation are shown on pathway maps including central metabolism with glycolysis, citric acid cycle, and amino acid biosynthesis (**Figure 1**), ascorbate metabolism (**Figure 2**), and the raffinose pathway (**Figure 3**), These figures compare the metabolic response of ‘Korona’ in roots and leaves to responses previously reported in diploid (Ås, Tingvoll, and Alta) genotypes (Rohloff et al., 2012). When examining the relative abundance of the metabolites (**Figure 4**, **Supplementary Figure S1**), one observes a distinctive complementary pattern in leaves and roots, likely reflecting differences in physiological function, as well as, source-sink relationships. By examining the relative fold-changes occurring as a function of cold treatment one can see a great deal in similarity in the response of several metabolites to cold (**Supplementary Figures S1** and **S2**). Central glycolytic metabolites (**Figure 1**) such as PEP, G6P, glucose, and TCA intermediates aconitate, isocitrate, succinate, and fumarate; were similarly altered in both leaves and roots. However, robust differences were seen in shikimate and the derived aromatic amino acids in roots and leaves. Strong sustained increases in two distinct compounds of the raffinose pathway (galactinol and raffinose) were found in both leaves and roots (**Figure 3**). In leaves rapid and sustained increases in proline, aspartic acid, and putrescine were observed; however, in roots increases were only transient. Starkly dissimilar changes (both abundance and kinetics) within the amino acids, and in phosphorylated and non-phosphorylated hexoses were observed when comparing roots and leaves. In general, leaf and root levels of compounds with osmotic functions such as monosaccharides (fructose, glucose, galactose, and mannose) and their phosphorylated counterparts (F6P, G6P, and M6P), were increased and partly maintained at higher levels throughout the cold acclimation period. This observation applies also to a range of amino acids (arginine, asparagine, aspartate, ß-alanine, glutamine, *N*-acetyl-serine, ornithine, phenylalanine, serine, and tryptophan). While increases in the ascorbate pathway-associated metabolite galacturonate was similarly strongly increased in roots and leaves; glucuronate, gulonate, galactonate, ascorbate, and dehydroascorbate were much more strongly increased in roots than leaves (**Figure 2**). While strong increases of the amino acids threonine, phenylalanine, and leucine occurred in roots and in leaves; changes in serine, asparagine, aspartate, methionine lysine, glutamine, histidine, and ornithine were very different. For example, ornithine was strongly accumulated in roots but decreased in leaves; while histidine was just the opposite.

**FIGURE 5:**
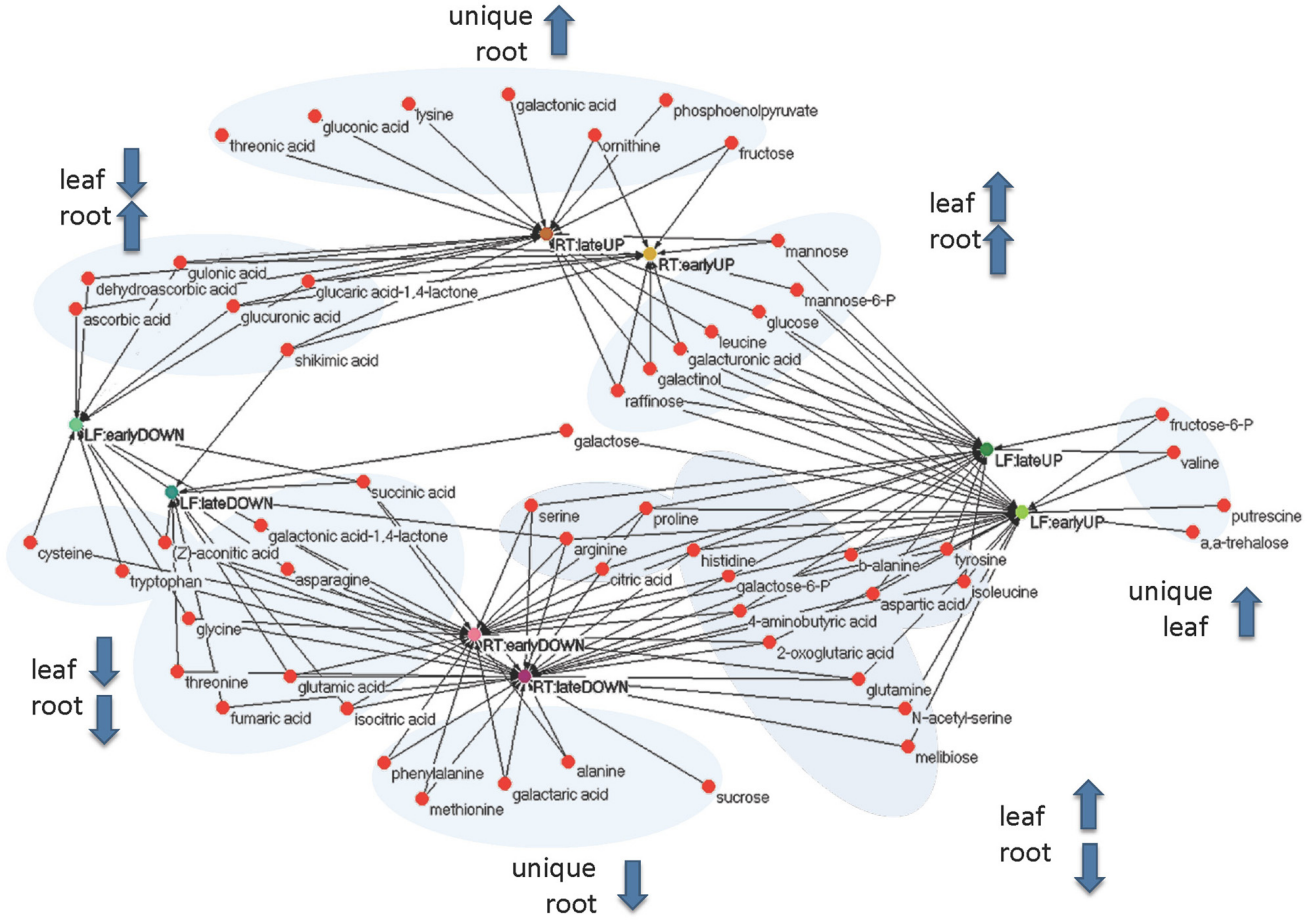
**Functional (unweighted) network of early (3 and 24 h) and late (72 and 240 h) metabolic responses upon cold treatment in leaf and root samples.** Rational for grouping in early and late responses is based upon principal component and clustering analysis (**Figure 6**).

**FIGURE 6:**
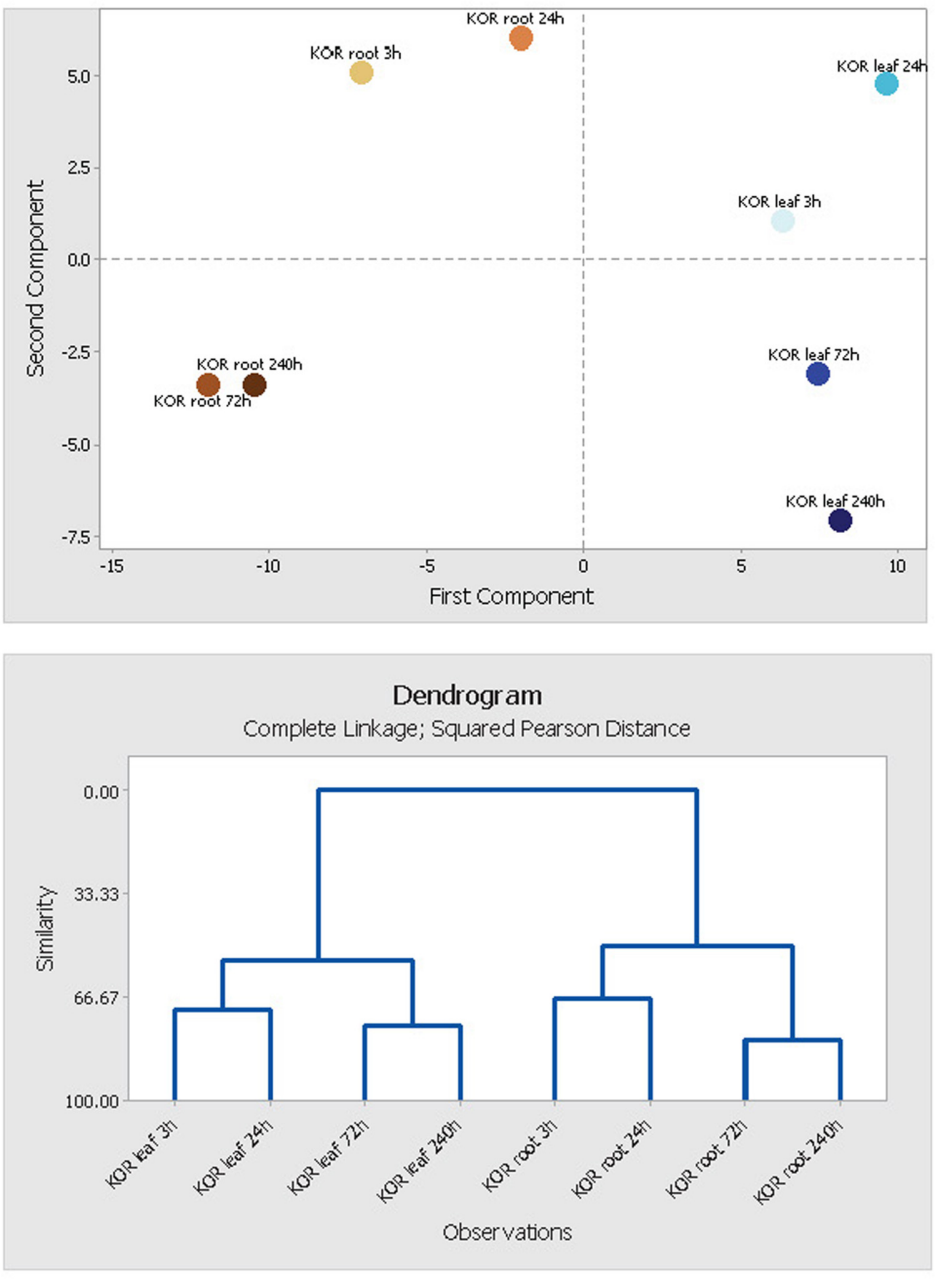
**Principal component analysis (PCA) of leaf and root metabolite profiles.** Segregation patterns of *F. ananassa* (KOR: cv. ‘Korona’) samples, harvested at different time points after cold treatment (0, 3, 24, 72, 240 h). The PCA diagram is based on 160 identified metabolites and non-identified mass spectral tags, and represents components PC1 × PC2 plotted against each other. PCA and tree clustering clearly indicate close similarity of metabolite profiles from time points 3 h/24 h and 72 h/240 h, and thus support the use of time point combinations for **early** (3 h/24 h) and **late** metabolic responses (72 h/240 h) being used in **Figure 5** (metabolite network).

In order to unify this analysis a network map was created to depict the alterations in metabolites in leaves and roots; grouping them in “early” and “late” responses (**Figure 5**). This map was supported by the demonstration of the similarity of responses by principal component analysis and by clustering analysis (**Figure 6**), where the data were clearly clustered into “early” (3 and 24 h) or “late” (72 or 240 h) responses. This allows a clear illustration of the metabolites having similar or different alterations in roots and leaves and in early versus late responses. For example, the intersection of common early responses to cold, in both leaf and roots, includes increases in raffinose, galactinol, galacturonic acid, and mannose-6-P; while the late increases in common include these (with the exception of mannose 6-P in the roots) plus glucose and leucine. Likewise, the early decreases common to both roots and leaves included, asparagine, cysteine, tryptophan, glycine, galactonic acid-1, 4-lactone, succinate, and aconitate. Interestingly, a significant number of metabolites were altered differently in roots and shoots. While ß-alanine, tyrosine, isoleucine, aspartic acid, serine, arginine, proline, histidine, glutamine, galactose-6-P, 4-aminobutyric acid, 2-oxoglutaric acid, *N*-acetyl-serine, citric acid, and melibiose were up in leaf (early or late); these all declined in the roots (either early or late). Metabolites uniquely accumulated in leaves included fructose-6-P, valine, putrescine, and α,α-trehalose; while metabolites uniquely accumulating in the roots included, gluconic acid, lysine, galacturonic acid, ornithine, and fructose.

### Modulation of Protein Expression in *Fragaria* under Cold Acclimation

Leaf samples of cold-treated *F. ananassa* ‘Korona’ from the 0, 24, and 240 h time points, were subjected to 2D gel protein separation and subsequent LC–MS/MS analysis of selected spots. Twenty-one spots were selected and successfully identified by mass spectroscopy. These included the most significantly different spots as well as several which appeared not to be significantly changing under any conditions. Sampling was avoided in the region containing RuBisCO LS because of possible contamination and since that spot was overloaded, it was not quantitatively evaluated. A representative 2D gel is presented in **Supplementary Figure S2**, indicating 21 distinct differentially regulated single spots that were identified (**Table 1** and see supportive details in **Supplementary File S2**).

**Table 1:** Proteins from 2DE identified by mass spectroscopy.

Spot ID	Identification	GenBank#	*Arabidopsis thaliana* homolog	Peptide sequences			Fold Change	
					
				Fv	Ros	Probability	24 h	240 h
5	Glycine cleavage system H protein	XP_004300260.1	AT1G32470	11	8	1	1.05	1.99
110	Chlorophyll a/b binding protein	XP_004293579.1	AT2G34430	33	26	1	1.77	1.82
403	RCA, RuBisCo activase	XP_004307478	AT2G39730	25	46	1	-1.39	1.93
1504	26S protease regulatory subunit 6A	XP_004302387.1	AT3G05530	2	2	1	1.77	1.21
1606	ATP synthase	ADY15336.1	ATCG00120	34	36	1	1.11	1.25
2004	ATP Synthase Delta-subunit	XP_004290445.1	AT4G09650	15	2	1	1.62	1.16
2208	Glyoxalase 1	XP_004287450.1	AT1G67280	9	10	1	2.09	1.21
2405	Glutamine synthase	XP_004288594.1	AT1G66200	35	35	1	-1.19	1.04
2507	Eukaryotic initiation factor 4A-9-like	XP_004291063.1	AT1G54270	3	11	1	-4.82	-3.08
3007	Cytochrome b6-f complex	XP_004293472.1	AT4G03280	11	11	1	-5.11	-1.42
3109	Chlorophyll a-b binding protein CP26	XP_004300672.1	AT4G10340	20	18	1	1.86	4.35
3502	Phosphoglycerate kinase, chloroplastic-like	XP_004306579.1	AT1G56190	45	44	1	1.69	1.92
3715	Annexin Dl-like	XP_004288206.1	AT1G35720	24	16	1	-1.09	1.49
3801	CLPC/ HSP93-V	XP_004297496.1	AT5G50920	35	33	1	-1.03	1.18
4004	Cytochrome b6-f complex iron-sulfur subunit	XP_004293472.1	AT4G03280	6	5	1	1.13	1.12
4010	SMALLER WITH VARIABLE BRANCHES (SVB)	XP_004300077.1	AT1G56580	9	8	1	-1.53	-6.52
4403	Flavanone 3-Hydroxylase	AAU04792.1	AT3G51240	3	3	0.9999	-2.41	-1.78
4817	ATP-dependent zinc metalloprotease FTSH	XP_004291222.1	AT1G50250	8	8	1	-1.32	1.67
7408	ADH2 Alcohol dehydrogenase (class-3)	XP_004290003.1	AT5G43940	3		1	-1.09	-1.42
8004	Auxin-binding protein ABP19a-like	XP_004287628.1	AT5G20630		4	0.9994	-1.98	-2.25
8005	Ribulose bisphosphate carboxylase small chain	XP_004303137.1	AT1G67090	15	12	1	1.34	-1.06

*GenBank gi and accession codes corresponding to the 21 SSP after LC-MS/MS and analysis using *Fragaria* (Fv) and Rosaceae (Ros) database. Spot numbers refer to those in **Supplementary Figure S2**. The number of peptides matched and probability of correct protein identity (*q*-values) are shown. The *Arabidopsis* homolog was obtained by blasting the EST sequences. The fold changes (after cold treatment) were calculated by using the average (*n* = 3) of the spot quantity values (normalized to all valid spots within a gel). Statistical significance and other supportive information is in **Supplementary File S2**.*

**FIGURE 7:**
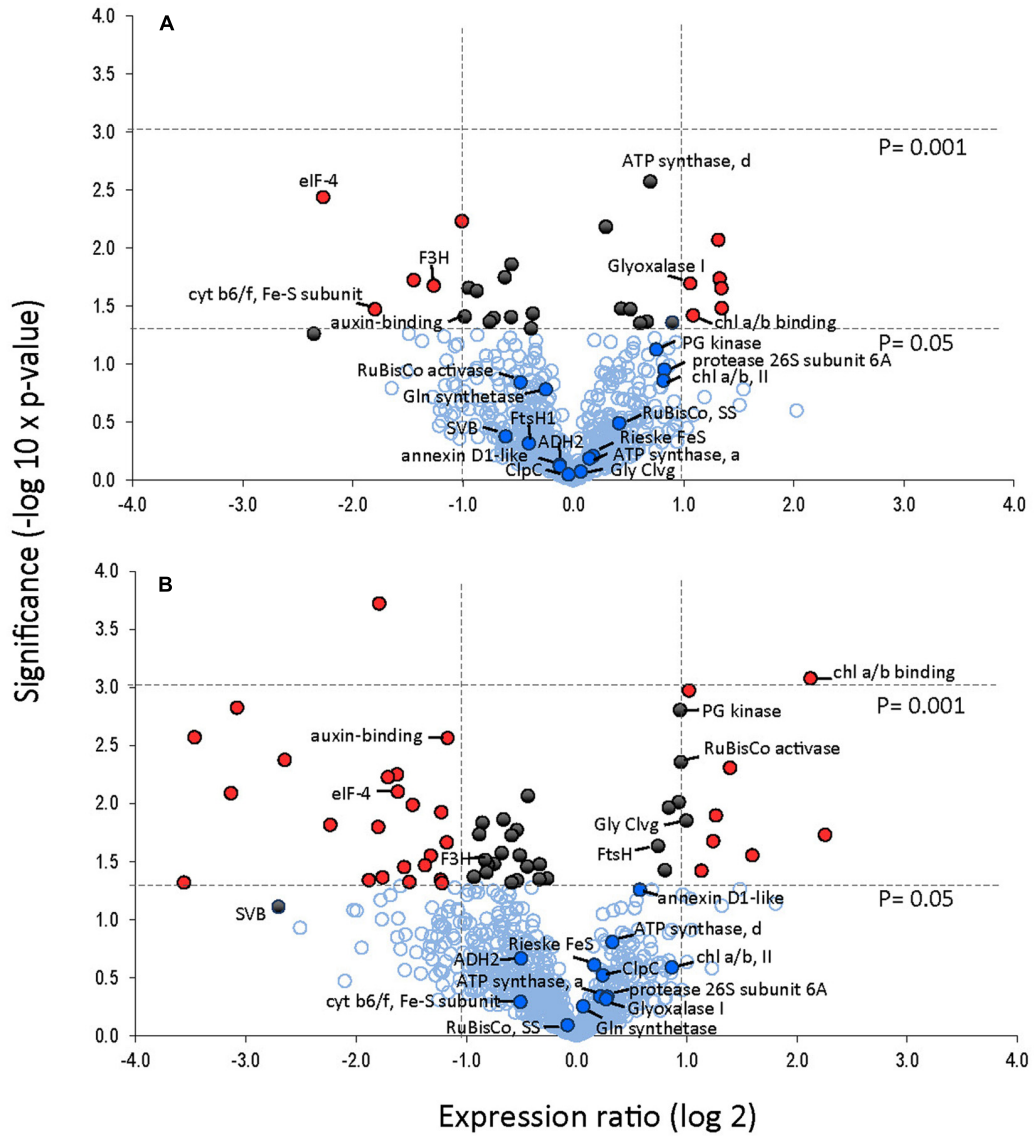
**Protein expression levels in leaves after 24 and 240 h of cold treatment **(A, B)**, respectively.** Volcano plot was obtained by plotting the log_2_ ratio of mean values (24 or 240 h cold treatment over control) for the 845 matched 2DE spots against the negative log10 of the *p*-value from the Student’s *t*-test. Proteins that changed twofold or more with a significance of *p*-value <0.05 are indicated with red. Proteins that changed significantly (*p* < 0.05) but changed less than twofold are indicated in black. Identified proteins are indicated.

**FIGURE 8:**
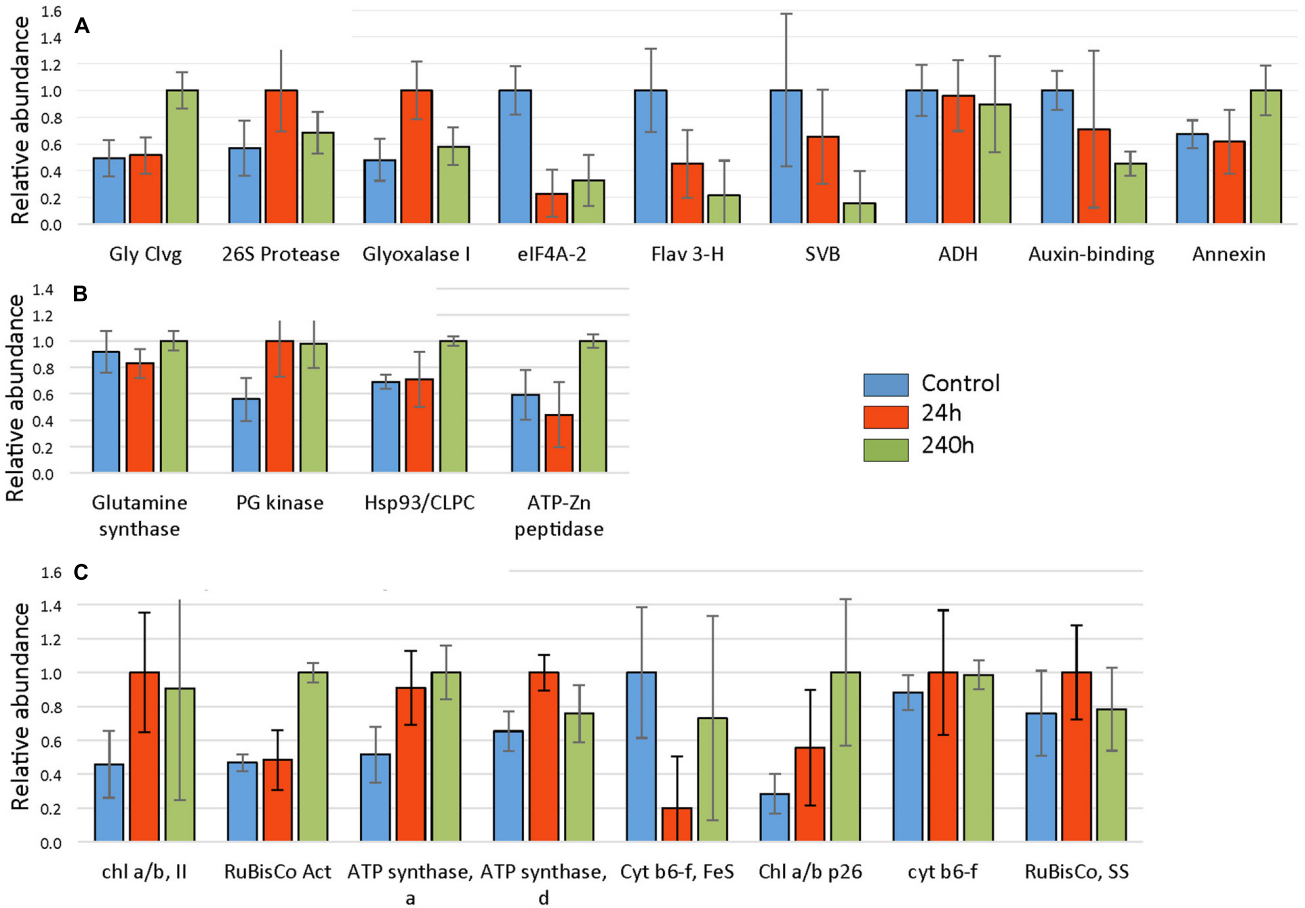
**Cold responses of twenty-one identified proteins.** Spot intensities were normalized to the highest expressor (i.e., the highest level of each protein was considered to be 1.0) and represent the average values of 3 gels (three replicate experiments) with error bars indicating standard deviations. Proteins were categorized into **(A)** non-chloroplastic, **(B)** chloroplastic metabolism, and **(C)** photosynthesis-related chloroplast proteins. Proteins identified and corresponding spot numbers were Gly Cleavage enzyme (SSP 0005), chl a/b, II (SSP 0110), RuBisCo Activase (SSP 0403), 26S protease regulatory subunit (SSP 1114), ATP synthase, alpha (SSP 1606), ATP synthase, delta (SSP 2004), Glyoxalase I (SSP 2208), Glutamine synthase (SSP 2405), eIF4A-2 (SSP 2507), Cyt b6-f, FeS (SSP 3007), Chl a/b p26 (SSP 3109), fumaryl acetase-like (SSP 3502), Hsp93/CLPC (SSP 3801), cyt b6-f FeS (SSP 4004), Flavanone 3 hydroxylase (SSP 4403), ATP-Zn peptidase, (SSP 4817), unknown, stress related (SSP 6711), alcohol dehydrogenase (SSP 7408), Auxin-binding (SSP 8004), RuBisCo, SS (SSP 8005).

**FIGURE 9:**
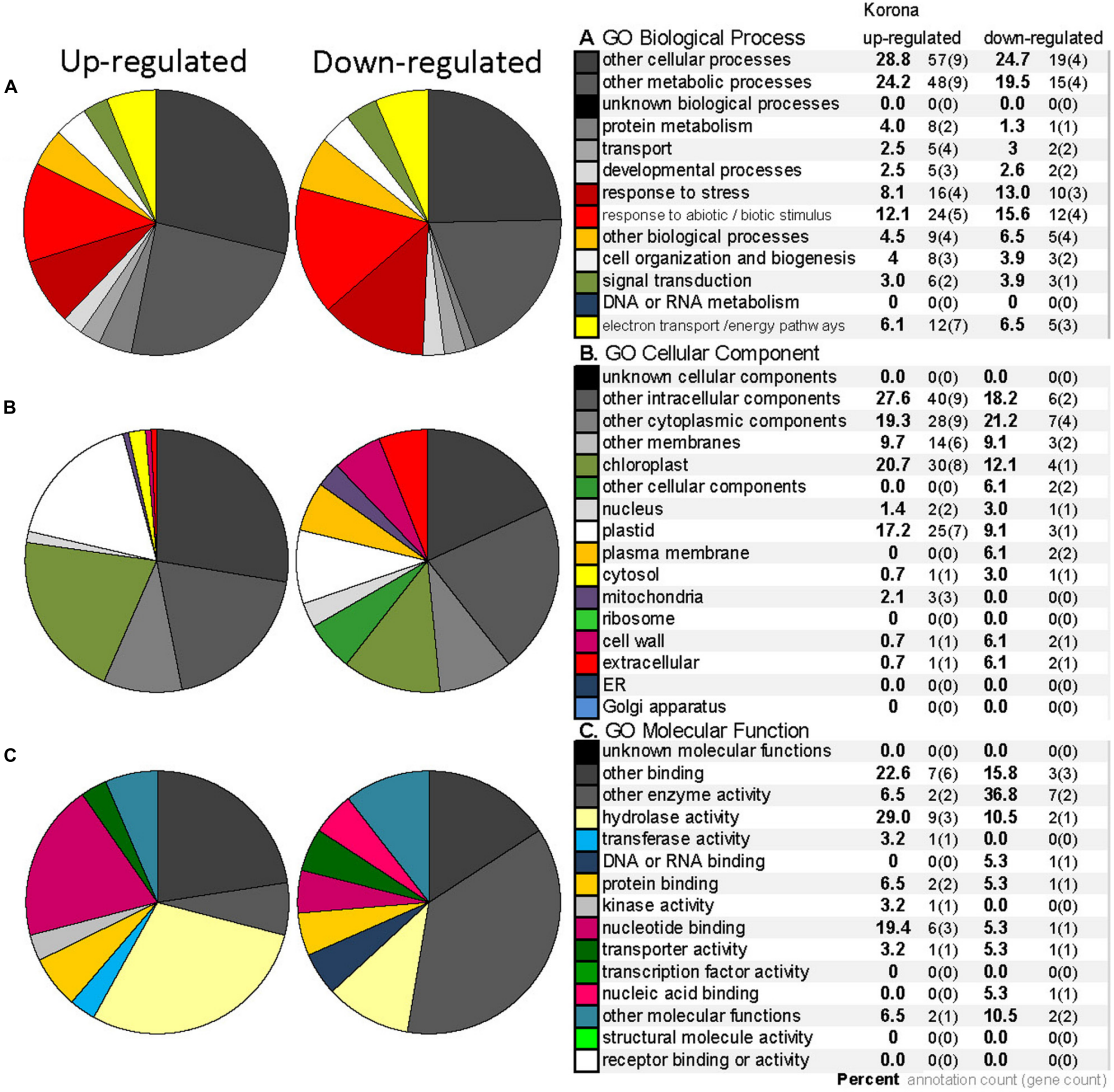
**Gene Ontology (GO) annotation for the differentially expressed proteins from 2DE analysis (homologous to *Arabidopsis* genes) in *F. ananassa* ‘Korona.’** GO categories for Biological Process **(A)**, Cellular Component **(B)**, and Molecular Function **(C)** for the differentially expressed proteins that were up-regulated (10 spots) or down-regulated (11 spots) greater than 1.5 fold after treatment at 2°C at either 24 or 240 h (listed in **Table 1**). Legend includes the percent (bold) next to number of annotations and number genes included within each category.

A total of 845 spots were matched in all nine gels, with 50/845 (4.6%) changing in spot quantity (*t*-test < 0.05). There were about threefold more down-regulated than up-regulated spots. The quantitative changes of the twenty-one spots which were successfully identified and their statistical significance (**Figures 7** and **8**, **Supplementary File S2**) and their gene ontology is shown (**Figure 9**). Many chloroplast-associated proteins (twelve of the 21 identified) were affected during cold acclimation. The identified photosynthetic proteins (**Figure 8C**) changed a maximum of about fivefold, with the chlorophyll a/b binding protein showing the greatest changes. The chloroplast ATP synthase gene (delta subunit) increased 1.6 fold at 24 h and returned to control levels at 240 h. RuBisCO SS protein did not change significantly throughout the cold acclimation period (RuBisCO LS was not quantitated), while RuBisCO activase increased significantly upon cold treatment after 240 h. Two cytochrome b6-f genes differed slightly with one (SSP 3007) decreasing sharply after 24 h, but returning to starting levels after 240 h in cold. In contrast, the other cytochrome b6-f FeS (SSP 4004) did not change significantly at all. Chloroplast metabolic proteins (**Figure 8B**) all showed maximal levels at 240 h of cold. The significant up-regulation of the ATP-dependent Zn peptidase (at 240 h) is of interest because of its essential role in thylakoid formation and the removal of damaged D1 precursors in monomeric photosystem II reaction center complexes. The chloroplast import chaperone Hsp93 levels also increased. Phosphoglycerate kinase, involved in both glycolysis/gluconeogenesis and CO_2_-fixation, appeared to increase by 24 h of cold and stayed elevated at the 240 h time point.

Proteins characterized as non-chloroplastic showed differing expression patterns in response to cold (**Figure 8A**). An auxin-binding protein (function unknown), and eIF4a-2 both decreased significantly. The helicase eIF4a-2, important in translation, was strongly decreased in response to cold treatment. The eIF4a-2 is known to be post translationally modified by phosphorylation with observed lateral shifts in 2DE gels (Webster et al., 1991; Gallie et al., 1997; op den Camp and Kuhlemeier, 1998). The observed decrease most likely represents a change in phosphorylation state. The decrease of the F3H, a key enzyme of flavonoid biosynthesis in plants, indicates a distinct down-regulation of secondary metabolism in strawberry leaves ‘Korona’ upon cold exposure. Both the glycine cleavage enzyme H and the unknown function stress-related protein (SVB) increased strongly upon cold treatment after 240 h. The putative glyoxalase I, which has potential detoxification functions involving sulfhydryls and methylglyoxal (a by-product of the glycolytic pathway) increased transiently at 24 h.

**FIGURE 10:**
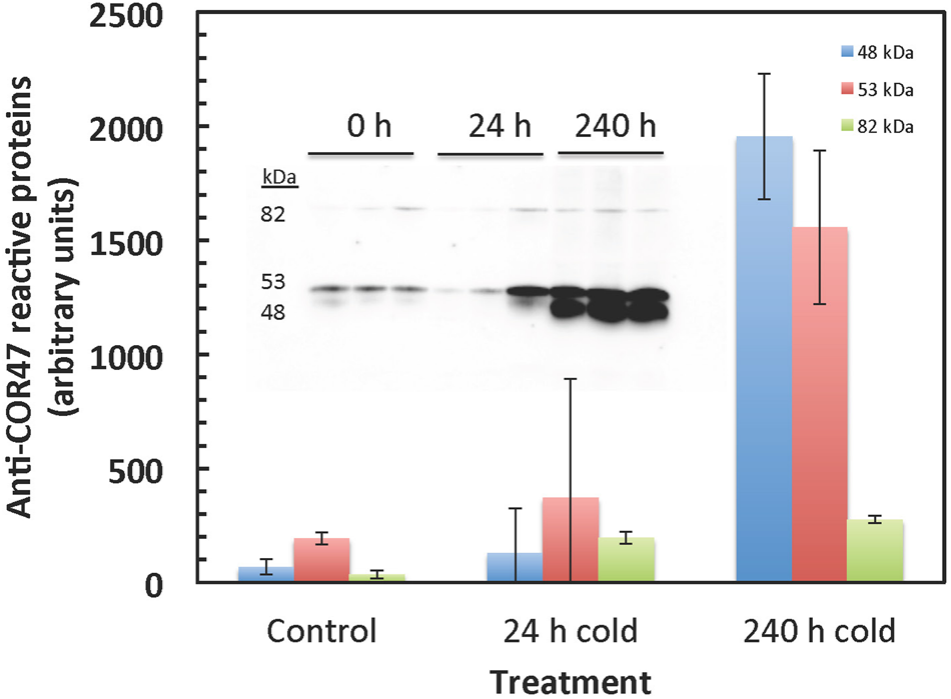
**COR47-reactive bands strongly increase in response to cold.** COR47 protein levels increase with extended cold treatment. Western blots were probed with antibody raised against *Arabidopsis* COR47. Two major bands were detected at 53 and 49 kDa, likely corresponding to the *Fragaria* COR47 homolog. The 53 kDa and the 49 kDa bands likely correspond to the phosphorylated and non-phosphorylated proteins, respectively. The upper minor protein (82 kDa) is probably an often observed aggregate of COR47. The insert shows the gel data used to calculate the averages and standard deviations.

Dehydrin protein levels were measured (**Figure 10**) in order to verify expected cold responses in leaf tissue of *F. ananassa* ‘Korona’ with regard to the well-characterized up-regulation of genes encoding dehydrins (Alsheikh et al., 2005). Gels probed with antibodies which specifically recognize the *Arabidopsis* dehydrin, COR47, revealed a significant increase in protein levels of a 53 kDa band, designated as FaCOR47 due to its cross reactivity to the antibody and its appropriate mass. Likewise, another antibody-reactive band (48 kDa) was highly expressed similarly upon cold treatment. This lower band likely represented the non-phosphorylated form of FaCOR47 (Alsheikh et al., 2005). The higher mass but minor band of 82 kDa is likely an aggregate of COR47 often detected in such blots. Increased transcript levels of COR47 (**Figure 10**), and other dehydrins (**Supplementary File S3**) support this observed protein response to low temperatures.

### Microarray and qRT-PCR

Transcripts from leaves were analyzed by microarray. Genes with an adjusted *P*-value less than 0.05 were considered as statistical significantly differentially expressed, and were categorized as transcripts associated with cold and general stress, metabolic, transport, development and organogenesis, photosynthesis, translation, transcription, signaling, and secondary metabolism. A total of 248 differentially expressed genes, altered by more than 1.5 fold are shown in **Supplementary File S3**. Cold-responsive transcripts dominated the significantly changing transcripts with twenty-four accumulated by 24 or 74 h of cold. Among these were several dehydrins, heat shock cognates, cold shock protein, galactinol synthase, glutathione peroxidase, and catalase. With the exception of two FTSH genes (involved in cleavage and recycling of the reaction center-binding D1 protein) all transcripts identified in the photosynthesis related category were significantly decreased in response to cold. Transcripts related to translation were all either insignificantly changed or decreased in response to cold as were the secondary metabolism transcripts. F3H exemplified this strong decrease in transcripts related to secondary metabolism.

**FIGURE 11:**
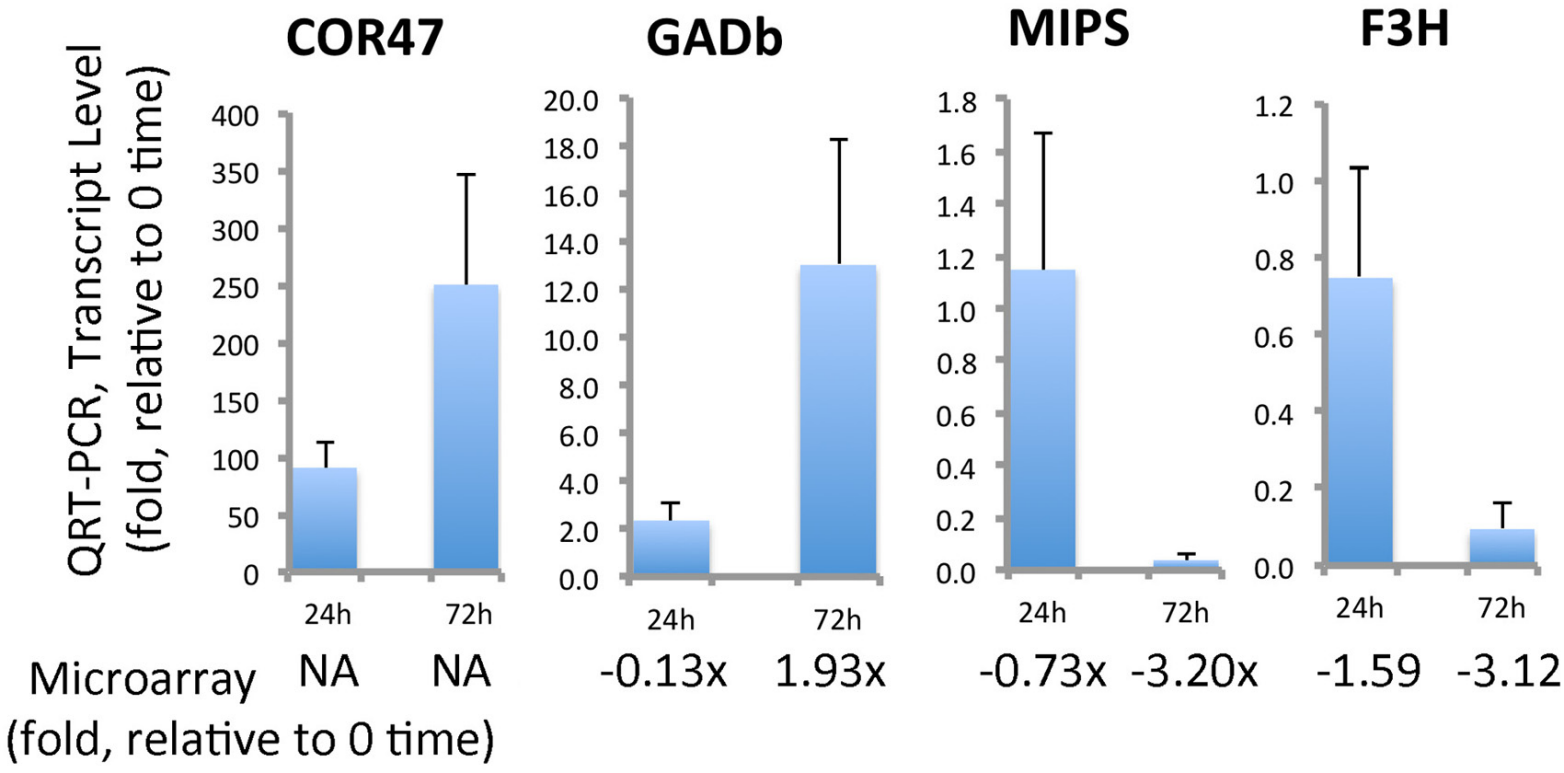
**Quantitation of selected cold-responsive transcripts.** COR47 (positive control gene for cold stress), GADb (glutamate decarboxylase, ortholog b), MIPS (myo-inositol-1-phosphate synthase), and F3H (flavonone-3-hydroxylase). Data represent average values of technical replicates, error bars indicate standard deviations. Primers utilized for this qRT-PCR analysis can be found in **Supplementary File S4**. The qRT-PCR is compared to fold-changes obtained from the microarray analysis (**Supplementary File S3**).

qRT-PCR analyses was conducted for several transcripts to verify and further quantitate results found in the microarray analysis (**Figure 11**). Transcripts for GADb (glutamine decarboxylase) increased approximately 13-fold by 72 h. F3H and MIPS decreased approximately 20-fold and 10-fold, respectively. Several dehydrins were found by microarray to be strongly accumulated, but COR47 was not identified in ‘Korona.’ Since protein levels of COR47 (**Figure 10**) were strongly induced, corresponding transcript levels were examined, indicating they were also strongly accumulated in response to cold, increasing 250-fold by 72 h.

## Discussion

### General Comments

According to the categorization of Shinozaki and Yamaguchi-Shinozaki (2006), homeostatic processes in plants upon low temperatures comprise the induction of regulatory and functional proteins, the latter being involved in the biosynthesis of compatible solutes and osmoprotectants (Kurz, 2008), membrane transport mechanisms (Lundmark et al., 2006), detoxification, and macromolecule protection. Due to the nature of the adopted global “omics” approach in this study, metabolic and biochemical changes are discussed with respect to primary metabolism.

### Modulation of Photosynthesis and Carbohydrate Metabolism under Cold Acclimation

One important coping mechanism for low temperature stress in plants is the reduction of the photosynthetic capacity to prevent situations where light energy harvested by the leaves might be in excess of what can be processed by the photosystems. Cold tolerant crops have been reported to maintain leaf respiration and photosynthesis rates during exposure to cold (Yamori et al., 2009, 2010, 2011) as opposed to more cold intolerant lines showing strong decrease in photosynthesis. Light intensity, cold intensity and duration, nutrition and metabolic interactions between organelles can influence the cold response of plants in terms of molecular changes associated with photosynthesis. In addition, diverse responses to cold temperatures are observed for different plant types, such as woody and herbaceous plants, indicating different strategies are utilized for coping with photosynthetic adjustments during low temperature exposure. However, despite the different strategies observed in diverse plants for coping, it is recognized that optimal photosynthesis requires a balance between the rates of carbon fixation in the chloroplast and cytosolic sucrose synthesis. Gao et al. (2009) pointed out the significance of photosynthetic acclimation and chloroplast adaptation as essential processes to obtain frost tolerance in plants.

Light harvesting, CO_2_-assimilation and photosynthetic biomass production rely mainly on the factors of light, temperature, and water. The experimental set-up in our study with short-day, reduced light, and constant, low temperatures at 2°C established quite unfavorable conditions for photosynthesis to operate effectively. It is thus not surprising that both structural and functional elements, enzymatic reactions and in turn, metabolism in *Fragaria* were dramatically affected. Low temperatures leads to a reduction of photosynthesis (Campbell et al., 2007; Usadel et al., 2008), the decreased capacity of which was also suggested in our study by down-regulation of Calvin cycle steps (FBP) and depressed levels of transcripts encoding subunits (proteins) of LHC, PSII, and PSI (**Supplementary File S3**). Light stress potentially leads to damage of PSII and reduced catalase activity (Campbell et al., 2007), which is consistent with our findings of suppressed CAT3 gene expression, and significant up-regulation of genes encoding FTSH1 and FTSH8 peptidases which act under high light and/or cold stress in thylakoid formation and removal of damaged PSII reaction center D1 protein. The 2DE analysis further confirmed the early (24 and 72 h) increase in transcriptional response of FTSH1 (ATP-Zn-peptidase; **Figure 8**) with significantly increased protein levels observed at 10 days. In addition, homeostatic recovery of photosynthesis and acclimation was further suggested through transcriptional increase of RuBisCO activase (RCA) after 72 h, followed by enhanced protein levels of RCA at 10 days, as well as with enhanced levels of two chlorophyll a/b binding proteins (LHCB5 and LHB1B2; **Figure 8**).

### Regulation of Osmolytes

The carbohydrates and their metabolic products establish an essential group of osmolytes showing generally increased levels in plant tissues upon exposure to low temperatures. Important sugars found in our study, where the concentration was drastically altered (>2.5-fold), comprised pentoses (lyxose, xylose), hexoses (fructose, glucose, sorbose, mannose, galactose, 1,6-anhydro-β, D-glucose) and disaccharides (sucrose). Furthermore, the raffinose pathway (**Figure 3**) was clearly affected resulting in increased levels of galactinol and raffinose, together with higher abundances of trisaccharides (melezitose), polyols (mannitol, myo-inositol, inositol conjugate form 2, 3, and 4) and phosphorylated sugars (F6P, G6P, Man6P, Gal6P; **Supplementary File S1**). Cold-induced changes in metabolite pools of soluble sugars in leaves have been described in earlier findings (Mattana et al., 2005; Yano et al., 2005; Korn et al., 2008), and the significance of both phosphorylated sugars (Kaplan et al., 2004; Gray and Heath, 2005) and the raffinose pathway (Cook et al., 2004) has been stressed. Similar metabolic shifts of soluble sugars have also been reported for below-ground tissues (Equiza et al., 2001; Bourion et al., 2003; Hekneby et al., 2006). Recently, raffinose pathway-derived structures have been shown to be involved in plant protection upon oxidative stress (Nishizawa et al., 2008). By 72 h of cold, alteration in the levels of galactinol (greater than 10-fold increase) and raffinose (fivefold increase) were substantial (**Figure 3**). It was interesting that at this same time point the MIP3 (myo-inositol-3-phosphate synthase) transcript levels (by microarray, **Supplementary File S3** or by qRT-PCR, **Figure 11**) had strongly decreased. It should be noted that while the drastic increase of hexose phosphates observed is associated with targeted biosynthesis of compatible solutes, these compounds exert a greater ROS scavenging capacity (e.g., F6P > fructose) compared to non-phosphorylated sugars as recently reported (Spasojević et al., 2009), possibly serving in roles both as stabilizing osmolytes and as ROS scavengers to contribute to cryoprotection.

### Stress-related Proteins, Antioxidants, and Detoxification Systems

An expected regulation of disaccharides upon cold such as the osmolyte trehalose found in *Arabidopsis* (Cook et al., 2004) was not found in our experiments, though a transient increase after 72 h occurred. The decrease in sucrose concentrations in roots, in combination with highly increased phosphorylated sugars in both roots and leaves (**Figure 1**) corroborate starch breakdown patterns described by Kaplan et al. (2007), leading to a rapidly increasing hexose-phosphate pool and finally, fructose and glucose levels. Induced mobilization of soluble sugars is further underscored by the up-regulation of transcripts involved in starch catabolism (**Supplementary File S3**), such as α-glucan water dikinase (SEX1), α-amylase (AMY3), disproportionating enzyme 2 (DPE2), and the maltose transporter (MEX1). In a different context, the long-term cryopreservation of *Fragaria* meristems (Caswell and Kartha, 2009), sucrose and in some cases, glucose have been shown to be the ideal sugars in vitrification solutions (Vysotskaya et al., 1999; Suzuki et al., 2008) to prevent freezing damage of plant tissue. In our study, hexoses were found to be the favored soluble sugar, being highly up-regulated in leaf and root tissue over the 10 days cold period. Sucrose has been shown to be transiently up-regulated (Kaplan et al., 2007) after 48 h, and is involved in the regulation of cold acclimation in *Arabidopsis* during diurnal dark periods (Rekarte-Cowie et al., 2008).

### Differential Regulation of Amino Acid Metabolism in *Fragaria* Genotypes

Another group of important osmolytes acting in cold acclimation processes and establishment of freezing tolerance, are amino acids and typically, polycationic polyamines. Consistent with earlier low-temperature experiments in the model plant *A. thaliana* (Cook et al., 2004; Kaplan et al., 2007), levels of amino acids and polyamines were clearly (transiently) increased in leaves and roots (**Figure 1**), without obvious preference of their biosynthesis family or side chain polarity. Proline, previously described to be highly up-regulated in leaf tissue upon cold (Cook et al., 2004; Kaplan et al., 2007), was also strongly up-regulated here, but not in the root. It was suggested that it is unlikely that proline is the determining factor of frost tolerance (reviewed by Ruelland et al., 2009).

Putrescine (a diamine), strongly up-regulated in the leaf (transiently in the root) has been previously reported to serve in several biological functions related to cold treatment, such as acting as a compatible solute (Kaplan et al., 2004), as modulating antioxidant systems (Zhang et al., 2009), and signaling and controlling ABA levels (Cuevas et al., 2008). Other prominent amine structures (spermidine and cadaverine) were also detected in our study but due to low peak intensity and scattered data points in leaf and root samples, these metabolites were excluded from the list of selected metabolites. Unfortunately, glycine betaine (GB) the most significant osmolyte described in *F. ananassa* (Rajashekar et al., 1999) and other species (Kamata and Uemura, 2004; Parvanova et al., 2004), could not be detected by GC/TOF-MS due to the positive charge of the cationic functional group. GB has been shown to positively modulate physiological responses upon exogenous GB application (Rohloff et al., 2002; Einset et al., 2007a,b, 2008). However, in the present work potentially enhanced GB levels were not confirmed by alteration of levels of ethanolamine, a precursor which is channeled via several modifying steps to choline and potentially, GB biosynthesis (Caspi et al., 2014). Polyamines other than putrescine are likely to be positively regulated in cold acclimation. Methionine, a precursor of spermine/spermidine biosynthesis, was substantially increased in leaf tissue (but not in roots; **Supplementary File S1**). Transcript levels of *S*-adenosyl-methionine synthetase (MAT3), which leads to *S*-adenyl-L-methionine decreased slightly (**Supplementary File S3**); while transcripts for the *S*-adenosylmethionine decarboxylase proenzyme 2 (SAMDC2), which produces *S*-adenosyl-L-methionine, the major precursor of spermine/spermidine, increased approximately threefold; supporting the regulation of the polyamine route.

Aspartate and β-alanine were clearly affected as indicated by coordinately increased levels of both metabolites in leaves (**Figure 1** and **Supplementary File S1**) which agrees with other reports describing enhanced alanine and aspartate levels under cold treatment (Cook et al., 2004; Mattana et al., 2005; Mazzucotelli et al., 2006; Allan et al., 2008). An important contributor to stress responses, GABA (Shelp et al., 1999, 2012), was also strongly accumulated in leaves. Thus a distinctive pattern was obtained in roots and leaves with leaves showing a substantial sustained increase in all three compounds. Our leaf metabolic data was further confirmed through microarray and qRT-PCR of ‘Korona’ leaves, detecting highly increased transcript levels of glutamate decarboxylase (GADb; **Supplementary File S2**, and **Figure 11**) suggesting that while alternative pathways (leading to ß-alanine and GABA) from polyamines may be contributors, glutamate decarboxylase level is also likely an important contributor to the enhanced biosynthesis of GABA.

The branch of amino acid biosynthesis leading from pyruvate to the structures of isoleucine, leucine, and valine, have received little attention in contributions to cold-regulated metabolism in plants, though interestingly, their metabolic regulation throughout the 10-day cold period was modestly altered. It is interesting that amino acid biosynthesis may be regulated coordinately, but differently in roots and leaves. Following cold treatment, derivatives of α-ketoglutarate (proline, glutamate, glutamine, histidine, and arginine) all accumulate in leaves, but decrease in roots. Also the shikimate derived amino acids (tryptophan, tyrosine, phenylalanine) all decrease in roots in response to cold as do all those derived from 3-phosphoglycerate (cysteine, glycine, and serine).

### Cold affects Stress-related Proteins, Metabolites, Antioxidant, and Detoxification Systems

Although derived from totally different routes and biochemical processes, the group of cryoprotective proteins, namely dehydrins, actually show similarities to compatible solutes based on their hydrophilic properties. However, their function is thought to be guided toward the surface of macromolecules (Ruelland et al., 2009) and enzymes (Reyes et al., 2008), membranes (Houde et al., 2004), and have a function in ion binding (Alsheikh et al., 2003). Since metabolite pools are not directly affected by the induction of dehydrins, results regarding their regulation are solely based on transcriptional and protein data. One of the most prominent cryoprotectants induced in plant systems exposed to sudden or gradual temperature decline, are members of the dehydrin family, e.g., COR47 (Alsheikh et al., 2005). A *Fragaria* COR47 homolog, was strongly increased at both protein and transcript levels by cold exposure (**Figures 10** and **11**). This strong increase in dehydrin levels found in ‘Korona’ is consistent with the strong correlation of dehydrins found with cold tolerance in *F. vesca* (Davik et al., 2013).

Secondary metabolites are a group of quite diverse compounds, and their expression levels are generally modulated under the influence of abiotic or biotic stress factors (Glombitza et al., 2004). Although GC/TOF-MS metabolite profiling is only capable of detecting a few phytochemical structures of this group (flavonoids and benzenoids), data from transcriptional and 2DE analyses indicated a cold-induced, down-regulation of the flavonoid pathway (**Supplementary Files S2** and **S3**). One of the most prominent candidates in the route leading to flavones, flavonols, and anthocyanins, is the F3H, which was clearly down-regulated in ‘Korona’ based on qRT-PCR of F3H transcripts (**Figure 11**) and the detected protein levels (**Figure 10**). It is interesting that in cold-tolerant strawberry crowns, accumulation of the F3H protein (Koehler et al., 2012b) has been associated with cold tolerance. Moreover, a clear positive correlation between flavonol content and freezing tolerance has been reported (Korn et al., 2008). Thus, it is interesting that regarding the flavonoid pathway, ‘Korona’ appears to have characteristics of a less cold tolerant cultivar.

As reported by several authors, plant antioxidant systems are distinctly impacted by cold acclimation processes, leading to typically increased levels of ascorbate (Streb et al., 2003; Cook et al., 2004; Dai et al., 2009) but additionally to other components such as oxidized sugars (Cook et al., 2004). Ascorbate was rapidly and strongly increased in roots, but the accumulation was greatly delayed in leaves. Moreover, a similar pattern was also found for dehydroascorbate. Metabolic regulation of these compounds has to be seen in close relationship to the ascorbate–glutathione (ASC–GSH) cycle that is present in different subcellular locations within plant cells: in the cytosol, peroxisomes, mitochondria and chloroplasts (Chew et al., 2003). Generally, ROS accumulation occurs in plant tissue under chilling stress but also as a consequence of cold acclimation processes (Kamińska-Rożek and Pukacki, 2005; Einset et al., 2007a,b, 2008; Gülen et al., 2008; Zhang et al., 2008), due to decreased enzyme activity and thus, less efficient ROS scavenging capacity. This is illustrated by the strong down-regulation of the gene encoding the superoxide dismutase (**Supplementary File S3**). This enzyme is responsible for the dismutation of superoxide anion radicals O_2_^-^ to H_2_O_2_, which is further detoxified by ascorbate in an enzymatic reaction with ascorbate peroxidase (APX). APX was clearly down-regulated in peroxisomes (APX3) and chloroplasts (APX4). Furthermore, transcript levels of the (cytosolic) dehydroascorbate reductase (DHAR2) were drastically decreased, underscoring the reduced potential for re-cycling of the produced dehydroascorbate. The GSH peroxidase 6 (GPX6) was slightly up-regulated, suggesting an increase in the cycling of GSH to GSSG (glutathione disulfide). This is consistent with reports on enhanced peroxidase activity and levels in *F. ananassa* (Gülen et al., 2008). Other members of the glutathione-*S*-transferase family (GST) are involved in catalytic detoxification (Janda et al., 2003; Ezaki et al., 2004). Their action in catabolic detoxification processes under low temperature conditions in both leaf and root tissue has been addressed (Janda et al., 2003; Ezaki et al., 2004). Members of different subclasses of GSTs (GSTU, GSTF) found to be significantly induced upon cold treatment in the present study include GST6 and GST25. An up-regulation of GSTF4, 5, and 8, which are also induced by cold and other abiotic/biotic stresses (Thatcher et al., 2007; Dixon et al., 2009), was previously observed. These are involved in detoxification of compounds carrying sulfate, nitrile, or halide groups. Yet another biological process activated in plants under stress conditions, is the glyoxalase system, which also involves the use of reduced GSH. Protein levels of glyoxalase I were clearly increased after 24 h but fell back by 240 h (**Figure 8**). Our protein data supports earlier results where GLX1 has been suggested to be potentially cold-induced (Seki et al., 2001; Koehler et al., 2012b). Since levels of the cytotoxic methylglyoxal, which derives from triose phosphates are likely to be elevated under stress and cold conditions (Yadav et al., 2005), increased GLX1 protein levels point to the induction of methylglyoxal detoxification in *Fragaria*.

### Leaves as Surrogate of Subterranean Tissues for Cold Tolerance Biomarker Analysis

One aspect of this work was to discern whether sampling of leaf tissue could potentially be used as a surrogate for subterranean tissues (roots or crown) to predict overwintering success when screening large populations. To make that evaluation, a focus on common alterations in metabolite levels in both roots and shoots was made. Raffinose and galactinol as well as G6P and glucose, strongly accumulated in roots and leaves, and are thus candidates from major carbohydrate metabolism that should be further investigated as potential markers. Raffinose and galactinol changes in leaf and roots were highly positively correlated over the entire time course with Pearson correlation coefficients of 0.988 and 0.914; while glucose and G6P did not correlate as well over the time course due to temporal fluctuations in levels. Despite some commonalities in responses, examination of most other cold altered metabolites generally indicated significantly divergent responses in leaves and roots. Further, an examination of 2DE data showed that of the 200 most abundant spots in leaves, only 53% could be unambiguously matched to those in crowns (data not shown). Comparison of the protein composition of four varieties of octoploids previously characterized (Koehler et al., 2012b) revealed that of the approximately 900 spots identified, less than 30% were visible in ‘Korona’ leaf tissue. It is no surprise that the predominance of chloroplast and photosynthetic related proteins in the leaves (particularly the presence of RuBisCO) obscured many proteins. While appropriate antibodies may well get around the problem of RuBisCO interference; the metabolic (and protein to some extent) divergent responses in the leaves compared to roots suggests that analysis of overwintering success may well require analysis of the most relevant overwintering tissues, the crowns.

## Conclusion

Significant changes occur in *Fragaria* sp. under cold acclimation, at the transcriptional, protein, and metabolite level. The application of different multivariate-statistical calculations and combinatorial approaches emphasize the complexity of cold-induced perturbations in metabolite pools in plant biological systems. Changes comprise the induction of osmolytes and cryoprotective dehydrins, and photosynthetic acclimation. Antioxidant and detoxifications systems related to ascorbate cycling in strawberry plants are clearly cold-impacted. A comparison of the ‘Korona’ metabolic profiling to that of several *F. vesca* (diploid) varieties (Rohloff et al., 2012) demonstrates that the diploid genotypes had significantly higher ROS scavenging capacity due to increased metabolite abundances than those observed in ‘Korona.’ In addition, the regulation patterns of 3-cyanoalanine and potential interplay with ethylene metabolism observed in the diploids suggested novel roles in plant cold acclimation processes which further segregate the diploids from octoploids. The osmolyte proline could play a role in ‘Korona’; however, this study clearly emphasizes the significance of amines (putrescine) and possible roles of branched-chain amino acids (leucine, isoleucine, and valine). Based on both diploid *F. vesca* and octoploid *F. ananassa*, single metabolites from the raffinose pathway, amino acids, amines, oxidized sugars as well as dehydrins are potential biomarkers in the further validation of *F. vesca* crosses and octaploid breeding lines.

Differing phenotypes and genotypes have to be considered when interpreting results of cold-induced responses in plants. Annual plants such as *Arabidopsis* might have developed differing mechanisms in cold acclimation, biochemical routes, and temporal controls compared to biennial or perennial species. Most cold studies with *Arabidopsis* have been carried out at the vegetative stage, subsequent results are then interpreted to relate to the plants’ need to keep up with the negative effect of low temperature in the leaves, in order to potentially provide enough photosynthetic assimilates for flowering, seed setting; and an ultimately successful reproduction. Perennials like the *Fragaria* sp. likely have established distinctive strategies, not only to prepare for long-term freezing temperatures, but also to be positioned optimally for re-acclimation after winter. The summation of the data described here illustrates the multigenic contributions to cold tolerance and the possibility of combinatorial variations that may contribute to various degrees of cold tolerance. Overall, ‘Korona’ appears to possess some characteristics of cold tolerance species (e.g., protection of chloroplast metabolism, increase in the protective dehydrin proteins, increases in ascorbate levels). But also, ‘Korona’ appears to have characteristics generally associated with less cold tolerant species (such as a clearly compromised response to oxidation stress; illustrated by decreases in SOD, APX, DHAR, and low levels of antioxidants such as ascorbate, as well as a decreased influence of the flavonoid pathway. The combinatorial effect may thus lead to the apparent moderate standing of ‘Korona’ amongst the range of cold tolerance in *Fragaria* sp. (Koehler et al., 2012b; Davik et al., 2013). The results thus support optimism for the successful breeding of plants with distinctive cold-tolerance traits to create enhanced cold tolerance in strawberry and other species.

## Author Contributions

GK, JR, RW, JK, AE, PW, AB, JD, MH, and SR all made substantial contributions to the work, participated in the revisions, and are accountable to specifics of work as follows. GK, SR; proteomics, RW, QRT-PCR; JR, JK, AE; metabolomics; JR, PW, AB; microarray. Major contributors to the writing were GK, SR, JR with all authors participating in descriptions of their unique contributions. JR, AB, SR, JD, RW, and MA all contributed to the design and interpretation of the experiments.

## Conflict of Interest Statement

This work was partially supported by a joint governmental grant and a commercial entity, Graminor Breeding Ltd. Dr. Muath K. Alsheikh an author on this paper is also employee of Graminor. Though this may appear to be a possible conflict of interest, there is nothing in this manuscript which was influenced by the company. The other authors declare that the research was conducted in the absence of any commercial or financial relationships that could be construed as a potential conflict of interest.

## Supplementary Material

Figure S1 **Heat Map.** Hierarchical clustering (means) using Pearson correlation is based on 160 identified metabolites and non-identified mass spectral tags (in rows) from leaves and roots of *F. ananassa* ‘Korona’ sampled at different time points after cold treatment (0, 3, 24, 72, and 240 h; in columns). Heat map visualization is based on log_2_(N) ratio of the normalized concentration levels to the corresponding median metabolite concentration of all samples. Blue indicates lower concentration levels of metabolites, and red indicates higher metabolite levels than the overall median.

Figure S2 **2D gel electrophoresis.** Representative 2D gel (24 h cold treatment) of leaf tissue extracts from *F. ananassa* ‘Korona,’ showing a high number of spots of which 21 distinct single spots are identified in the figure (see also **Figure 7** and **Supplementary File S2**).

FILE S1 **Metabolites identified by GC/MS-TOF.** Identified metabolites and as yet non-identified mass spectral tags of leaf and root samples from cold-treated *F. anananssa* ‘Korona.’ Additional data, previously published (Rohloff et al., 2012), is included to allow comparison of the octaploid ‘Korona,’ with previously published metabolic data from several diploid species. Data are average (*n* = 5) normalized responses of time points 3, 24, 72, and 240 h after exposure to 2°C and a non-cold treated control (t0). Log response ratios are centered to the median of t0. Metabolites represented by 2 or more alternative trimethylsilylated or methoxyaminated analytes were aggregated by summation of the normalized responses. Note that non-identified MSTs are indicated by an identifier code of the Golm Metabolome Database.

FILE S2 **List of identified proteins from 2DE.** Protein spot statistics and descriptions include GenBank gi and accession codes corresponding to the 21 SSP after LC-MS/MS and database analysis using Fragaria and Rosaceae database. The available best peptide sequence and (*n* = 3) of the corresponding *q*-values are listed. The Arabidopsis homolog was derived from blasting the EST sequences in TAIR. The percent identities matching and probability values are listed. The fold changes were calculated by using the average spot quantity values (normalized to all valid spots within a gel). Statistical analysis includes ANOVA-Tukey and performing an unpaired *t*-test.

FILE S3 **Microarray-based transcriptional analysis.** Differentially regulated genes in leaves of cold-treated *F. anananssa* ‘Korona’ from time points 24 and 72 h (0 h time point served as control). Data are shown as log_2_ fold change (compared to time 0). Those transcripts changing greater than 1.5 fold (and with *p* < 0.05) are highlighted. Genes are categorized into the following groups (sheets): A – Cold and Stress; B – Metabolism; C – Transport; D – Development and Organogenesis; E – Photosynthesis; F – Translation; G – Transcription; H – Signaling; I – Secondary Metabolism.

FILE S4 **List of qRT-PCR primers utilized for Figure 11**.
