# Supplementary figures and images for: Integrative “omic” analysis reveals distinctive cold responses in leaves and roots of strawberry, Fragaria × ananassa ‘Korona’
Source: Front Plant Sci. 2015 Oct 15;6:826. doi: 10.3389/fpls.2015.00826 (PMC4606020; doi:10.3389/fpls.2015.00826)

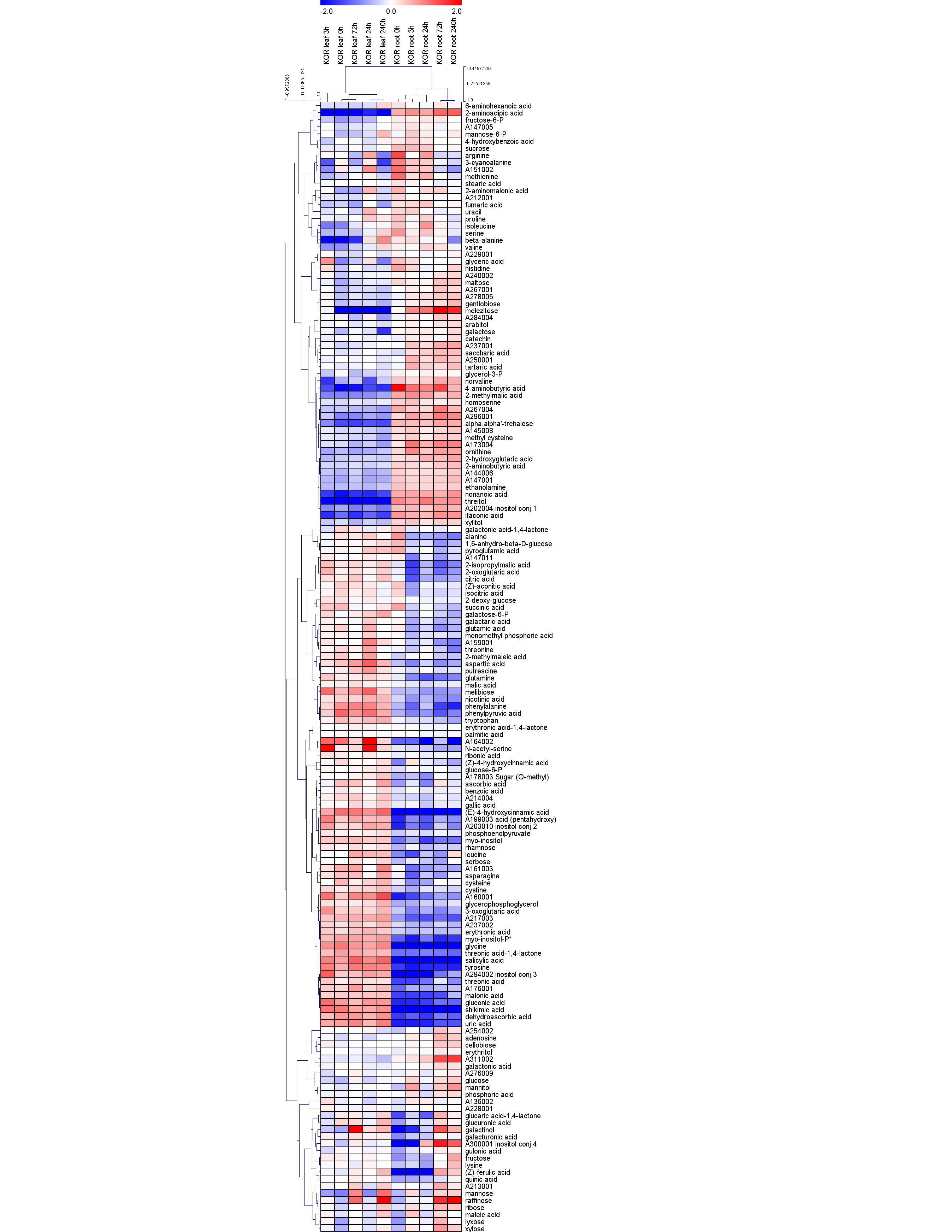

Supplement: Figure S1 — Heat Map. Hierarchical clustering (means) using Pearson correlation is based on 160 identified metabolites and non-identified mass spectral tags (in rows) from leaves and roots of F. ananassa ‘Korona’ sampled at different time points after cold treatment (0, 3, 24, 72, and 240 h; in columns). Heat map visualization is based on log2(N) ratio of the normalized concentration levels to the corresponding median metabolite concentration of all samples. Blue indicates lower concentration levels of metabolites, and red indicates higher metabolite levels than the overall median. [file Image_1.JPEG]

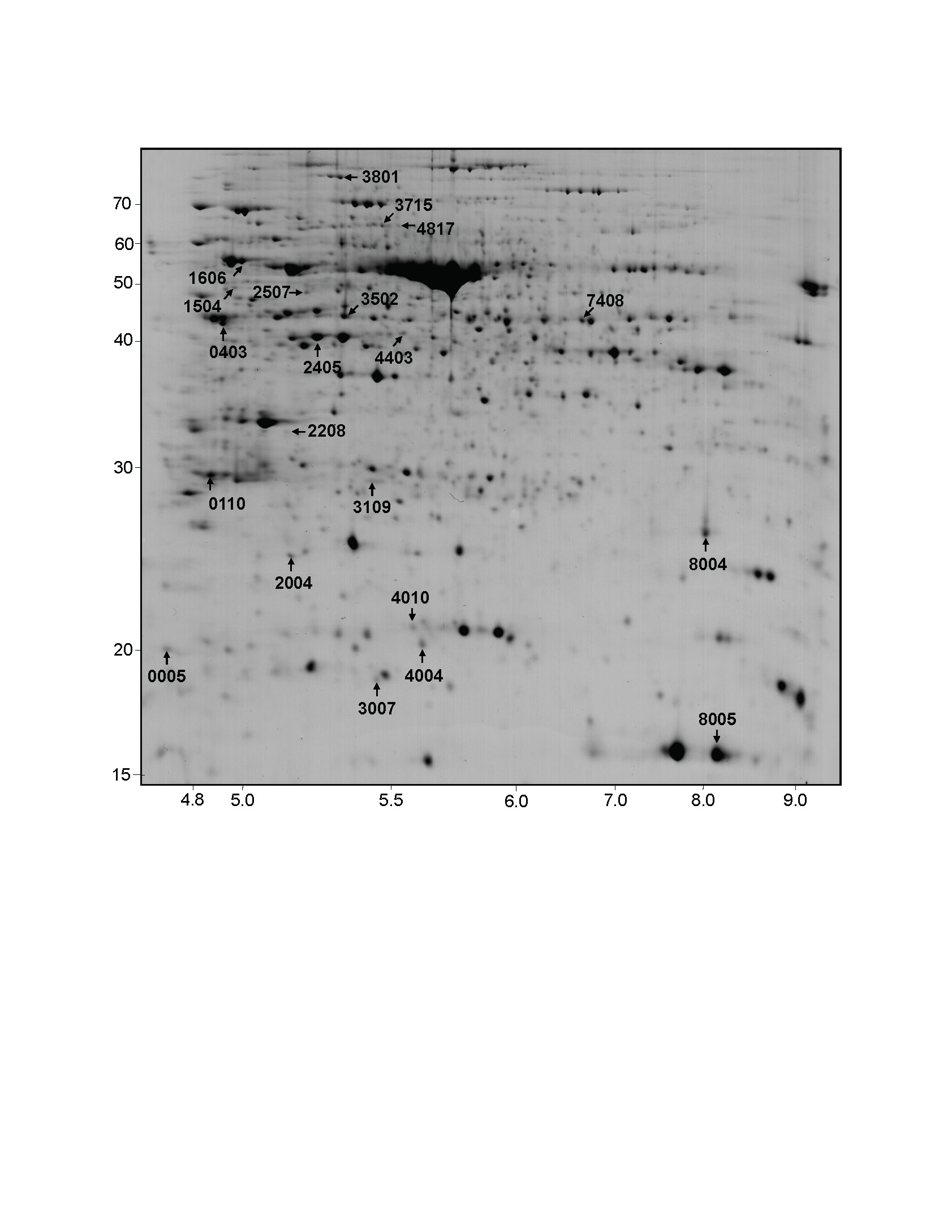

Supplement: Figure S2 — 2D gel electrophoresis. Representative 2D gel (24 h cold treatment) of leaf tissue extracts from F. ananassa ‘Korona,’ showing a high number of spots of which 21 distinct single spots are identified in the figure (see also Figure 7 and Supplementary File S2). [file Image_2.JPEG]
